# Genome-Wide Identification of AP2/ERF Transcription Factors in Cauliflower and Expression Profiling of the ERF Family under Salt and Drought Stresses

**DOI:** 10.3389/fpls.2017.00946

**Published:** 2017-06-08

**Authors:** Hui Li, Yu Wang, Mei Wu, Lihong Li, Cong Li, Zhanpin Han, Jiye Yuan, Chengbin Chen, Wenqin Song, Chunguo Wang

**Affiliations:** ^1^College of Life Sciences, Nankai UniversityTianjin, China; ^2^College of Horticulture and Landscape, Tianjin Agricultural UniversityTianjin, China

**Keywords:** AP2/ERF transcription factor (TFs), cauliflower (*Brassica oleracea* L. var. *botrytis*), salt stress, drought stress, expression profiling

## Abstract

The AP2/ERF transcription factors (TFs) comprise one of the largest gene superfamilies in plants. These TFs perform vital roles in plant growth, development, and responses to biotic and abiotic stresses. In this study, 171 AP2/ERF TFs were identified in cauliflower (*Brassica oleracea* L. var. *botrytis*), one of the most important horticultural crops in *Brassica*. Among these TFs, 15, 9, and 1 TFs were classified into the AP2, RAV, and Soloist family, respectively. The other 146 TFs belong to ERF family, which were further divided into the ERF and DREB subfamilies. The ERF subfamily contained 91 TFs, while the DREB subfamily contained 55 TFs. Phylogenetic analysis results indicated that the AP2/ERF TFs can be classified into 13 groups, in which 25 conserved motifs were confirmed. Some motifs were group- or subgroup- specific, implying that they are significant to the functions of the AP2/ERF TFs of these clades. In addition, 35 AP2/ERF TFs from the 13 groups were selected randomly and then used for expression pattern analysis under salt and drought stresses. The majority of these AP2/ERF TFs exhibited positive responses to these stress conditions. In specific, *Bra-botrytis-ERF054a, Bra-botrytis-ERF056*, and *Bra-botrytis-CRF2a* demonstrated rapid responses. By contrast, six AP2/ERF TFs were showed to delay responses to both stresses. The AP2/ERF TFs exhibiting specific expression patterns under salt or drought stresses were also confirmed. Further functional analysis indicated that ectopic overexpression of *Bra-botrytis-ERF056* could increase tolerance to both salt and drought treatments. These findings provide new insights into the AP2/ERF TFs present in cauliflower, and offer candidate AP2/ERF TFs for further studies on their roles in salt and drought stress tolerance.

## Introduction

Plant transcription factors (TFs) play vital roles in plant growth, development, and responses to various environmental stresses (Rashid et al., 2012; Shu et al., 2016). The APETALA2/ethylene-responsive element binding factor (AP2/ERF) superfamily is one of the largest groups of TFs (Nakano et al., 2006), which contain one or two AP2 domains with 60–70 conserved amino acid residues. The amino acid residues are all composed of a three-stranded anti-parallel β-sheet and an α-helix (Allen et al., 1998). The AP2 domain is essential for the activity of AP2/ERF TFs by binding *cis*-acting elements including the GCC box motif, the dehydration responsive element (DRE)/C-repeat element (CRT), and/or the TTG motif in the promoter regions of their target genes (Ohme-Takagi and Shinshi, 1995; Jofuku et al., 2005; Sun et al., 2008; Wang et al., 2015). The AP2/ERF superfamily can be divided into the AP2, ERF, RAV, and Soloist families according to the number of AP2 domains and presence of other DNA binding domains (Nakano et al., 2006). AP2 TFs contain two AP2 domains or a single AP2 domain that is similar to the AP2 domains in the double domain groups, whereas ERF family members contain a single AP2 domain (Nakano et al., 2006). The ERF family is further subdivided into the ERF and dehydration responsive element binding proteins (DREB) subfamilies on the basis of the similarities in amino acid residues of the AP2 domain. With the exception of a single AP2 domain, there is an additional B3 domain in the RAV family. A small group of TFs with a highly diverged single AP2 domain (AP2-like domain) and gene structure is known as the Soloist family.

The AP2/ERF superfamily was previously believed to only exist in the plant kingdom, but recent reports have indicated that AP2/ERF TFs are also present in protists and ciliates (Rashid et al., 2012; Licausi et al., 2013). The function and regulation of AP2/ERF TFs were deeply explored. The TFs from the AP2 family mainly function in the plant-specific regulation of growth and developmental processes (Li et al., 2013; Horstman et al., 2014; Kuluev et al., 2015), such as flower development (Aukerman and Sakai, 2003), leaf epidermal cell identity (Moose and Sisco, 1996), and seed growth (Jofuku et al., 2005). The TFs from the ERF and DREB subfamilies, both classified into the ERF family, are closely associated with responses to environmental stress. ERF subfamily TFs bind to the GCC-boxes and are involved in several hormone signaling pathways, such as the ethylene, jasmonic acid, and salicylic acid pathways (Fujimoto et al., 2000; Oñate-Sánchez and Singh, 2002; Mantiri et al., 2008). These TFs specifically participate in the regulation of defense responses against various biotic stresses, such as pathogen and disease stimuli (Zhao et al., 2012; Dong et al., 2015). Some of these TFs facilitate tolerance against environmental stressors, such as drought (Seo et al., 2010), salinity (Seo et al., 2010; Zhang et al., 2011), and freezing (Zhang and Huang, 2010). By contrast, DREB subfamily TFs bind to the DRE/CRT elements in stress-responsive genes. These TFs are mainly involved in plant tolerance against abiotic stresses, such as freezing (Ito et al., 2006; Fang et al., 2015), drought (Hong and Kim, 2005; Fang et al., 2015), heat (Qin et al., 2007), salinity (Hong and Kim, 2005; Bouaziz et al., 2013), and osmosis (Fujita et al., 2011). The number of TFs in the RAV family is relatively less compared with those in the AP2 and ERF families. Several RAV TFs also facilitate the regulation of target gene expression in response to ethylene, brassinosteroid, and biotic and abiotic stresses (Hu et al., 2004; Mittal et al., 2014, 2015).

Cauliflower (*Brassica oleracea* L. var. *botrytis*) is an important variant of *Brassica*. It is also one of important horticultural crops with high nutritional content and has been planted worldwide. In the past two decades, traditional genetic methods, such as hybrid breeding, have contributed considerably to the production of new cauliflower varieties that exhibit high yields and resistance against pathogenic bacteria. Meanwhile, the classical breeding technology is time-consuming and hard sledding. Current genetic engineering methods, such as TAILEN (Joung and Sander, 2013) and CRISPR/Cas9 (Shan et al., 2014) technologies, have exhibited immense potential to achieve the molecular improvement of various crops in short periods and high efficiency. However, the mechanisms underlying the regulation of cauliflower growth, development, and response to different environmental stresses remain poorly understood thereby hindering the breeding of cauliflower varieties with excellent traits. TFs from the AP2/ERF superfamily play important roles in diverse plant development, but these roles in cauliflower remain unknown.

In the present study, TFs from the AP2/ERF superfamily were identified according to the transcriptome data of cauliflower. Cluster, phylogeny and distribution of conserved motif analysis of the identified AP2/ERF TFs were conducted. A total of 35 AP2/ERF TFs were randomly selected from the phylogenetic tree and subjected to expression pattern analysis under salt and drought stresses. The AP2/ERF TFs closely associated with salt and/or drought responses were confirmed and the function of one of them was further identified by ectopic overexpression analysis.

## Materials and methods

### Plant materials and stress treatments

Homozygous cauliflower seeds were planted in soil under controlled conditions with a 16 h/8 h light/dark cycle at 25°C and 22°C, respectively. The 25-day-old seedlings were subjected to salt and drought stresses. Under each stress conditions, 15 seedlings were used. For the salt-stress treatment, 15 individual plants were irrigated with 200 mM NaCl. The leaves and roots of each plant were harvested 0, 4, 8, and 24 h after the treatment. The leaves and roots of five plants at each time point were pooled to form a biological replicate, and then frozen immediately in liquid nitrogen, and finally stored at −80°C. The seedlings subjected to drought-stress treatments underwent the same process, except that they were irrigated with 20% PEG 6000 instead of 200 mM NaCl.

### RNA isolation and qRT-PCR

Total RNAs from the samples of salt-stress and drought-stress treatments at each time- point were extracted using TRIzol reagent (Invitrogen, USA) in accordance with the manufacturer's instructions. RNAs free of contaminated genomic DNA were subjected to a reverse transcription reaction with Oligo (dT)_18_ primers. The first-strand cDNAs of these RNAs were used as templates for the qRT-PCR analysis, in which specific primer pairs were used. The *Actin* gene from cauliflower was selected as the internal control (Table S1). FastStart Universal SYBR Green Master (Roche, Germany) was used for qRT-PCR. The relative expression levels of each gene at different stress treatments were calculated by the comparative 2^−ΔΔCT^ method. Three biological replicates and three technological replicates were performed to ensure the reliability of quantitative analysis.

### Identification of AP2/ERF TFs

The high-throughput transcriptome data of the cauliflower were obtained (Accession number: PRJNA361430). All unigenes produced from the transcriptome data were annotated using the Blastx tool (Altschul et al., 1990) by aligning the non-redundant data (ftp://ftp.ncbi.nlm.nih.gov/blast/db/) and Swiss- Prot database (http://www.uniprot.org/downloads) with the parameters of expect value <1e-5 and with more than 80% coverage. In Blastx analysis, the nucleotide sequences were translated into the predicted proteins based on the standard genetic code. According to the functional annotation information of these unigenes, the putative AP2/ERF TFs were retrieved from cauliflower and further evaluated for the presence of AP2 domains by searching against the conserved domain database at NCBI. In addition, HMM search (http://www.ebi.ac.uk/Tools/hmmer/search/hmmsearch) was also conducted to further retrieve and identify the possible TFs containing AP2 domains in cauliflower (Finn et al., 2015). Some unigenes produced by the transcriptome sequencing did not contain the full coding regions of the genes. Thus, the putative cauliflower AP2/ERF TFs with incomplete coding sequences were conducted to clone the full coding regions through homologous cloning strategy.

### Phylogenetic tree construction

The deduced amino acid sequences of the AP2/ERF TFs were performed to multiple sequence alignment analysis using Clustal W set at default parameters (http://www.genome.jp/tools/clustalw/). An unrooted phylogenetic tree was constructed using the neighbor-joining method by the MEGA 6 program with the following parameters: bootstrap value of 1,000, Poisson correction, and pairwise deletion (Tamura et al., 2013).

### Conserved motif analysis

The conserved motifs in the cauliflower AP2/ERF TFs were identified by using the online motif finding tool MEME 4.11.2 (http://meme-suite.org/tools/meme) (Bailey et al., 2009). The parameters were as follows: 6–200 optimum width of amino acids, 25 maximum number of motifs, and 0 or 1 single motif in each sequence of the model.

### Expression pattern analysis

On the basis of the phylogenetic tree of all the detected AP2/ERF TFs, at least two TFs from each group or subfamily were selected to conduct expression pattern analysis under salt or drought stress. The relative expression levels of the randomly selected TFs at each time- point of salt- and drought- stress treatments were evaluated by qRT-PCR. The transcript expression profiles of the AP2/ERF TFs were determined using hierarchical cluster analysis with the package “gplots” of the *R* project according to the value of log_2_ (relative expression level of each transcription factor) (http://www.r-project.org/).

### Construction of expression vector and plant transformation

To further elucidate the function of AP2/ERF TFs under abiotic stresses, one TF (*Bra-botrytis-ERF056*) that showed significantly rapid responses to both salt and drought treatments was selected to conduct functional analysis. The full-length coding sequences of *Bra-botrytis-ERF056* with *XbaI and SacI* restriction sites were amplified by primers: ERF056-forward: 5′TCTAGAATGGAATCCAAGCCTCTCG3′ and ERF056-reverse: 5′GAGCTCTTATGATTCGGACAATTTGCTA3′. The PCR products were cloned into the pEASY-T1 vector and digested with *XbaI* and *SacI*. The digested products were sub-cloned into the pBI121 binary vector. The recombinant plasmid was transformed into *Agrobacterium tumefaciens* strain LBA4404 and then introduced into *Arabidopsis thaliana* ecotype Columbia (Col-0) via the floral dip method (Clough and Bent, 1998). T_1_ seeds of the transgenic plants were selected on MS medium containing 50 mg/L kanamycin. The phenotypes of homozygous T_3_ generations of the transgenic plants were observed by 200 mM NaCl and drought treatments, respectively.

## Results

### Identification of AP2/ERF TFs in cauliflower

On the basis of the cauliflower transcriptome data, 171 unigenes containing AP2 domains were obtained and annotated according to the AP2/ERF TFs in other reported *Brassica* plants or *Arabidopsis*. Except for five unigenes, the other 166 unigenes were annotated as the homologs of AP2/ERF TFs in other plant species. The five unnamed unigenes were then named as *Bra-botrytis-AP2/ERF-1* to *Bra-botrytis-AP2/ERF-5*. Sequence analysis indicated that 45 of the 171 unigenes did not contain the full coding regions of the corresponding AP2/ERF TFs. Subsequently, the full coding sequences of these 45 AP2/ERF TFs were cloned and sequenced. Finally, the full coding regions of each AP2/ERF TF were identified, and the amino acids of each of TF were deduced (Table S2). The length of these putative proteins was 91–589 aa. Among these TFs, 15 containing two AP2 domains were classified into the AP2 family. Inside the AP2 domain, nine TFs each contained a conserved B3 domain. These TFs were generated from the RAV family. Meanwhile, the 146 TFs each containing a single AP2 domain were classified further into the DREB (55 members) and ERF (91 members) subfamilies. The deduced amino acids of the AP2 domain from *Bra-botrytis-AP2/ERF-4* were distinct from those of the other AP2/ERF TFs, which was classified into the Soloist family.

### Phylogeny of AP2/ERF TFs in cauliflower

**Figure 1:**
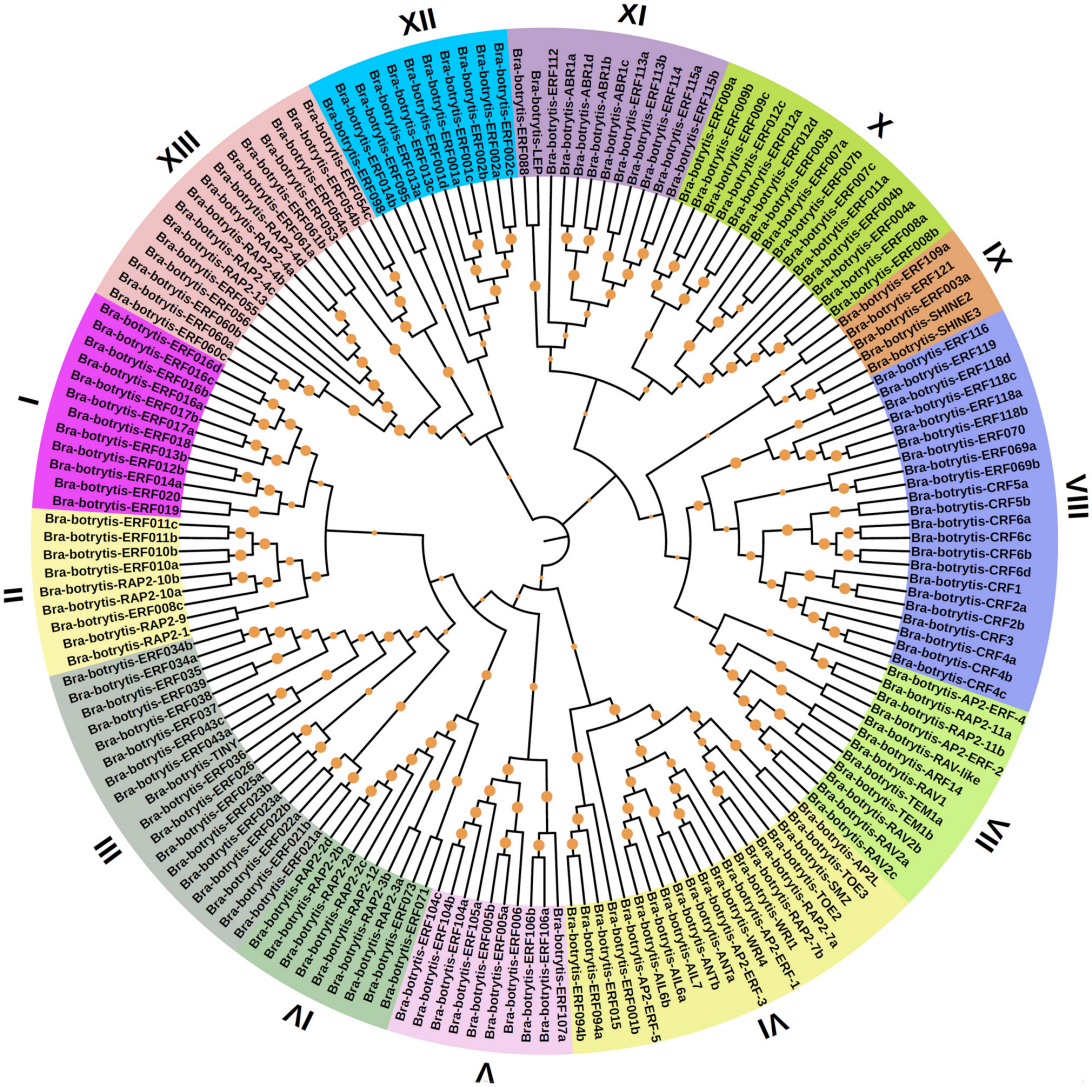
Unrooted phylogenetic tree of AP2/ERF transcription factors in cauliflower. Deduced full-length amino acid sequences were used to construct the phylogenetic tree using MEGA 6.0 software by a neighbor-joining method with bootstrap replicates of 1,000. Thirteen groups are highlighted in different colors. Bootstrap values over 80% were showed by dots with different size.

To confirm the classification and evolutionary relationships of the AP2/ERF TFs in cauliflower, the full-length sequences of the putative proteins were aligned and conducted to phylogenetic tree analysis. All of these AP2/ERF TFs could be classified into 13 clades (Figure 1). Group I contained 12 TFs, most of which contained motif-1, motif-2, motif-3, and motif-10, except *Bra-botrytis-ERF014a, Bra-botrytis-ERF019*, and *Bra-botrytis-ERF020*. Group II included 9 TFs, and nearly all TFs contained motif-1, motif-2, motif-3, motif-4, and motif-10 in their proteins. Group III comprised 18 TFs, and motif-1, motif-2, motif-3, motif-4, and motif-16 were detected in almost all these TFs. Groups I, II, and III were considered to be under the DREB subfamily. Group VI contained 20 TFs. A large proportion of the TFs in this group holding two AP2 domains were classified into the AP2 family. Group VII contained 12 TFs. Seven motifs (motif-1, motif-2, motif-3, motif-4, motif-5, motif-9, and motif-12) were detected in this group. Most of the TFs in this group belonged to the RAV family. Group XIII contained 16 TFs. The TFs of this group were also belonged to DREB subfamily. The TFs in other clades, except *Bra-botrytis-AP2/ERF-4*, were classified into the ERF subfamily (Figure 1, Figure S1).

### Conserved motifs of the AP2/ERF TFs

**Figure 2:**
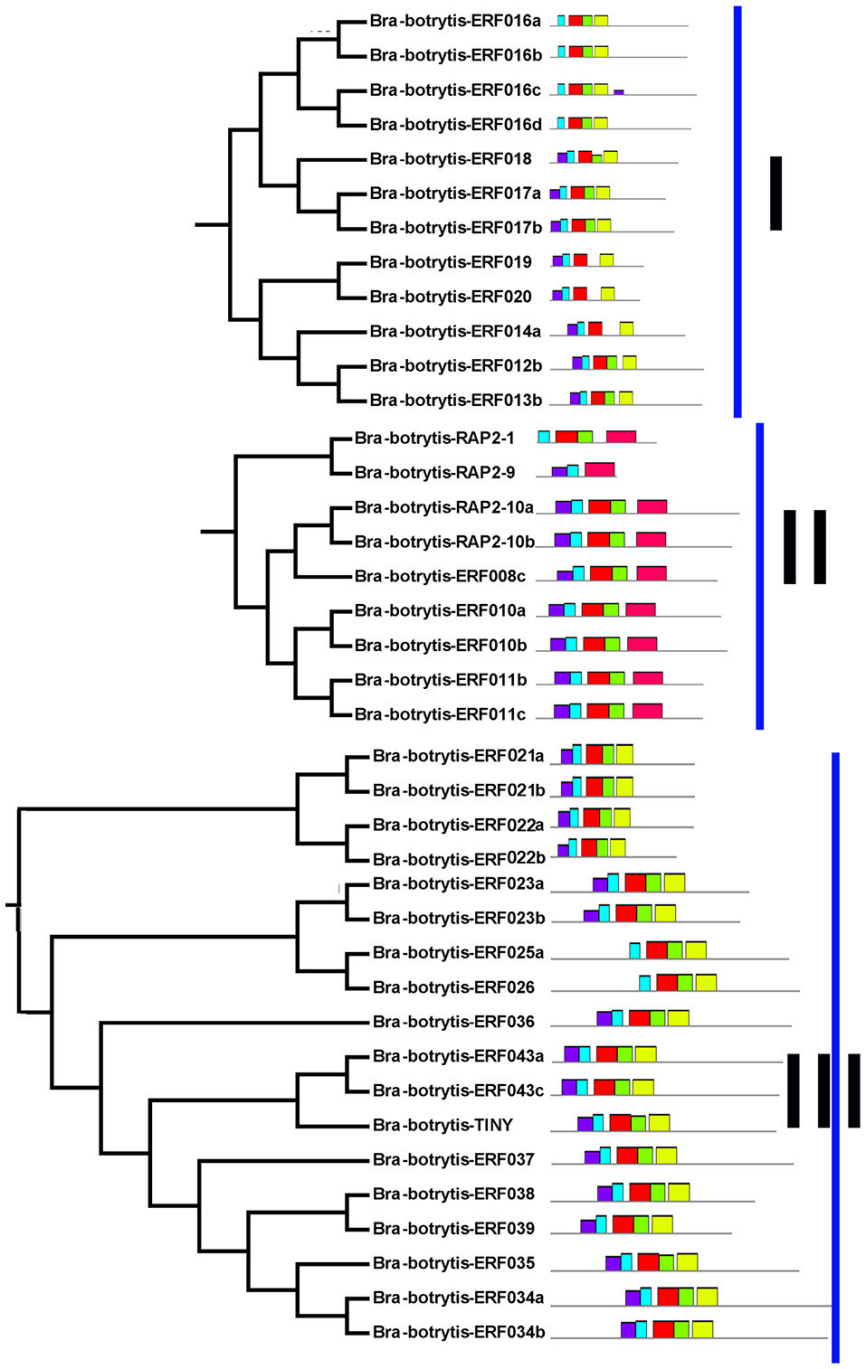
Distribution of conserved motifs within different groups of AP2/ERF Transcription factors in cauliflower. TFs (except Bra-botrytis-098) from groups I, II, III, and XIII belong to the DREB subfamily. Group VI-2 belongs to the AP2 subfamily. The majority of group VII belongs to the RAV subfamily. Members of other groups belong to the ERF subfamily. Different motifs are highlighted in different colors.

**Figure 3:**
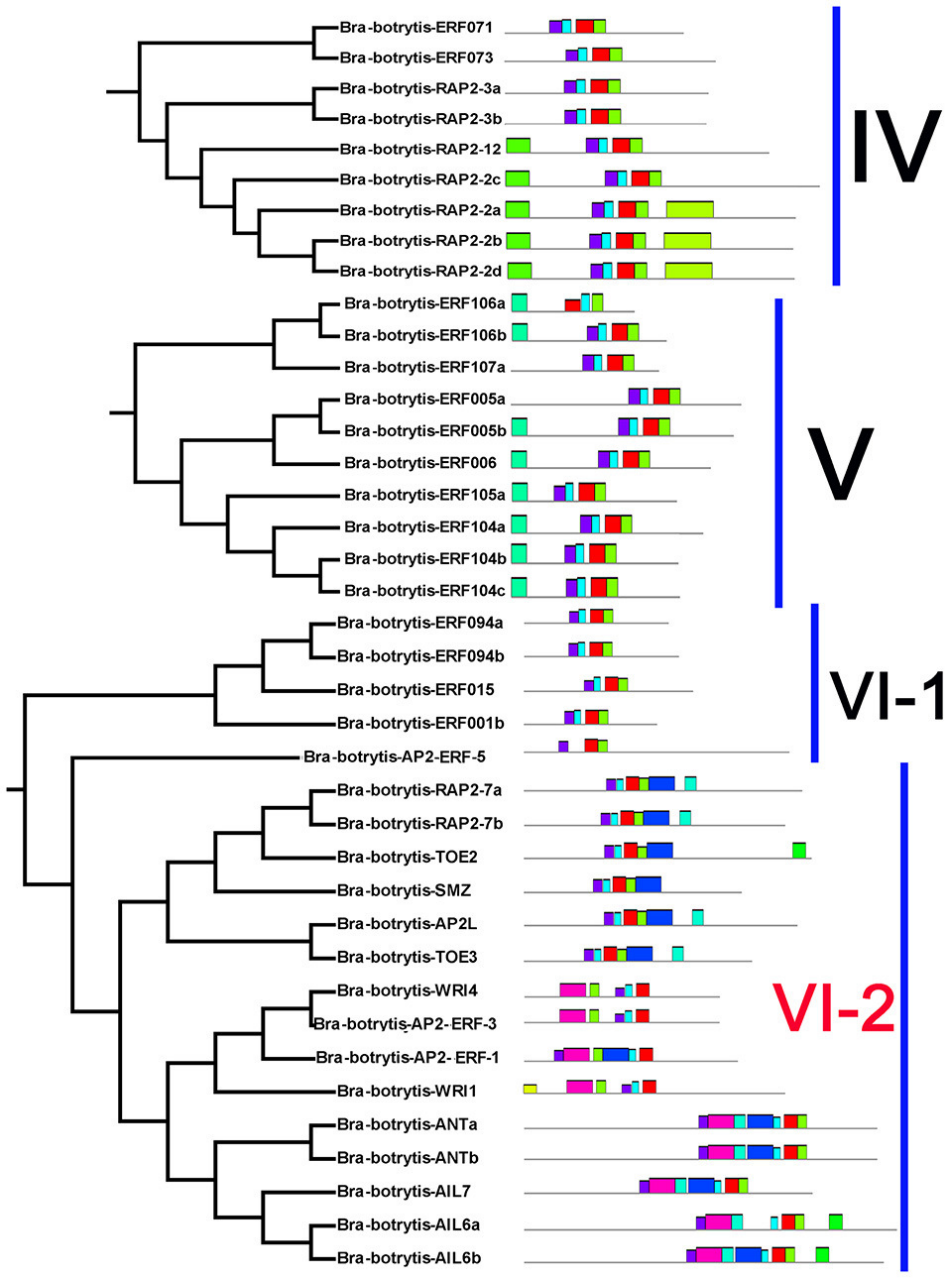
Please refer Figure 2 caption.

**Figure 4:**
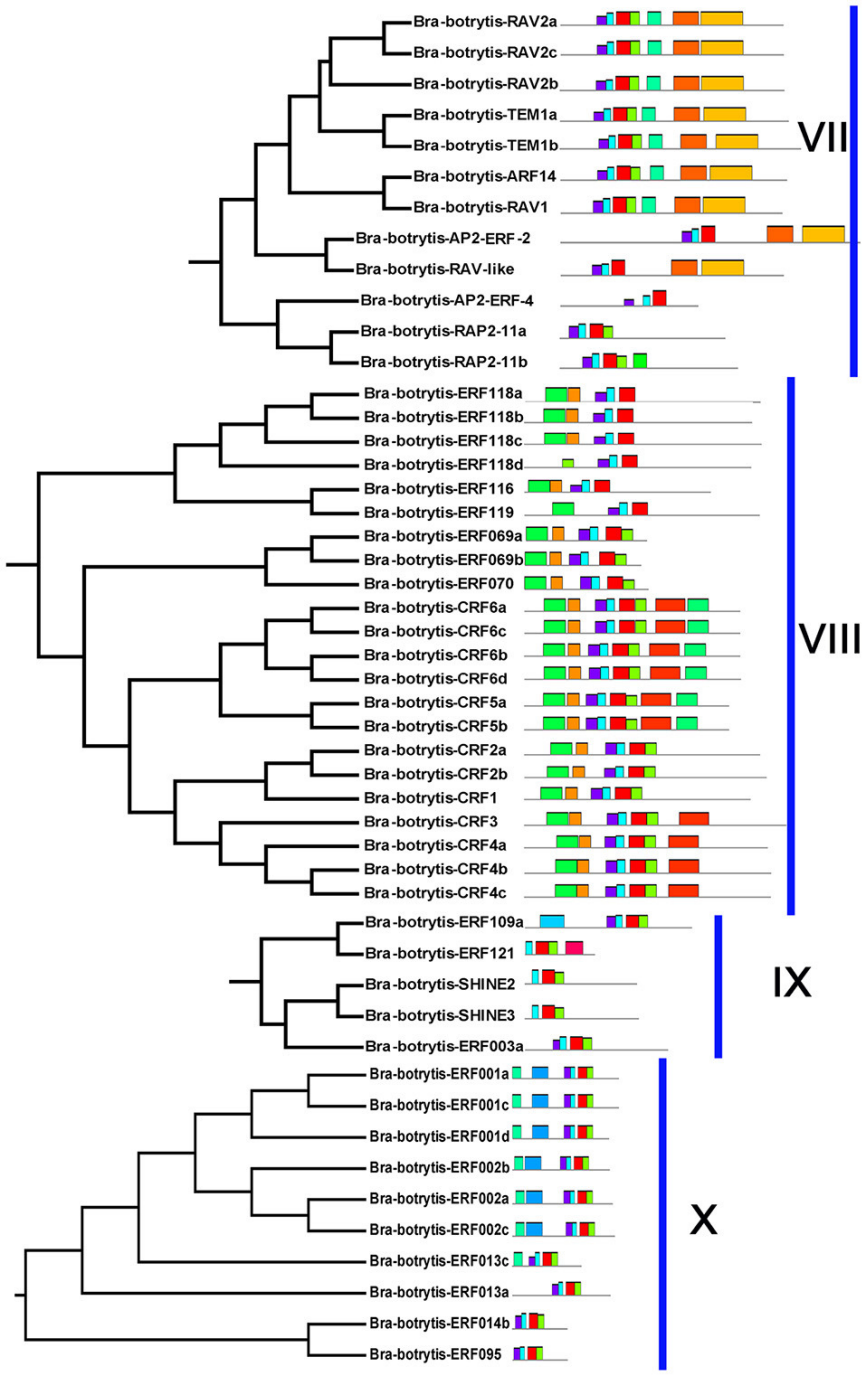
Please refer Figure 2 caption.

**Figure 5:**
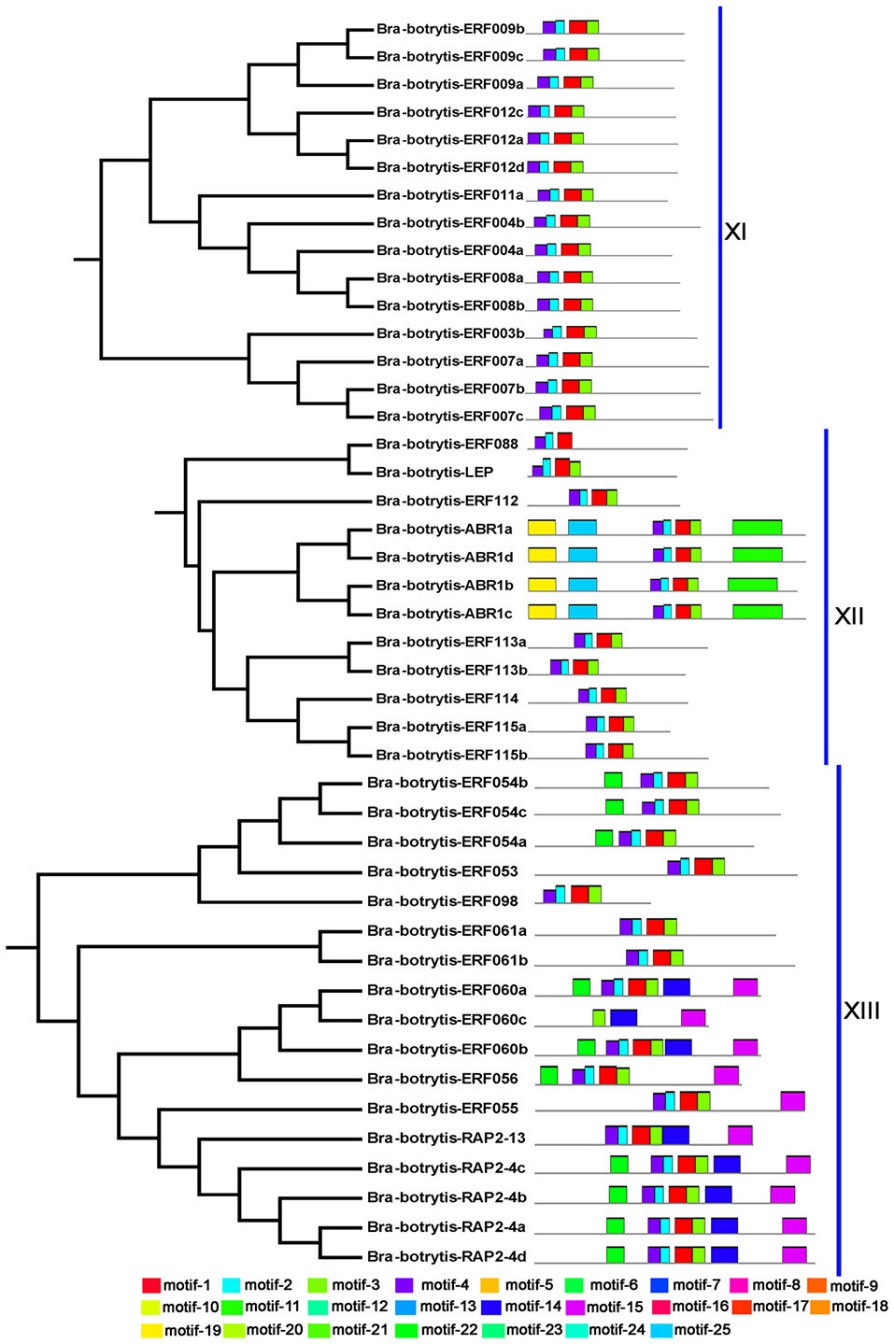
Please refer Figure 2 caption.

The conserved motifs in the AP2/ERF superfamily proteins of cauliflower were discovered by the MEME tool. A total of 25 conserved motifs were detected (Figures 2–5, Figure S1). Motif-1, motif-2, motif-3, motif-4, motif-8, and motif-24 were in the AP2 domain regions, among which motif-1, motif-2, motif-3, and motif-4 were detected in nearly all AP2/ERF proteins. Motif-5 and motif-9 were in the regions of the B3 domain. Proteins containing these two motifs were all classified into the RAV family. Other motifs were divergent among different groups or subgroups (Figures 2–5). Motif-6, motif-17, motif-18, and motif-23 were only detected in group VIII. Motif-7 was found only in group VI, which contains many TFs from the AP2 family. Motif-10 specifically existed in groups I and III. The TFs of these two groups were all classified into the DREB subfamily. Motif-11, motif-13, motif-16, and motif-19 were detected only in groups XII, X, II, and XII, respectively. Motif-14 and motif-15 were specific in group XIII. In addition, motif-12 was shared in groups V, X, and VII. Motif-20 and motif-21 were detected only in group IV. Motif-22 was mainly detected in groups XIII and VI. Motif-25 mainly existed in group XII.

### Expression profiling of AP2/ERF TFs under salt stress

**Figure 6:**
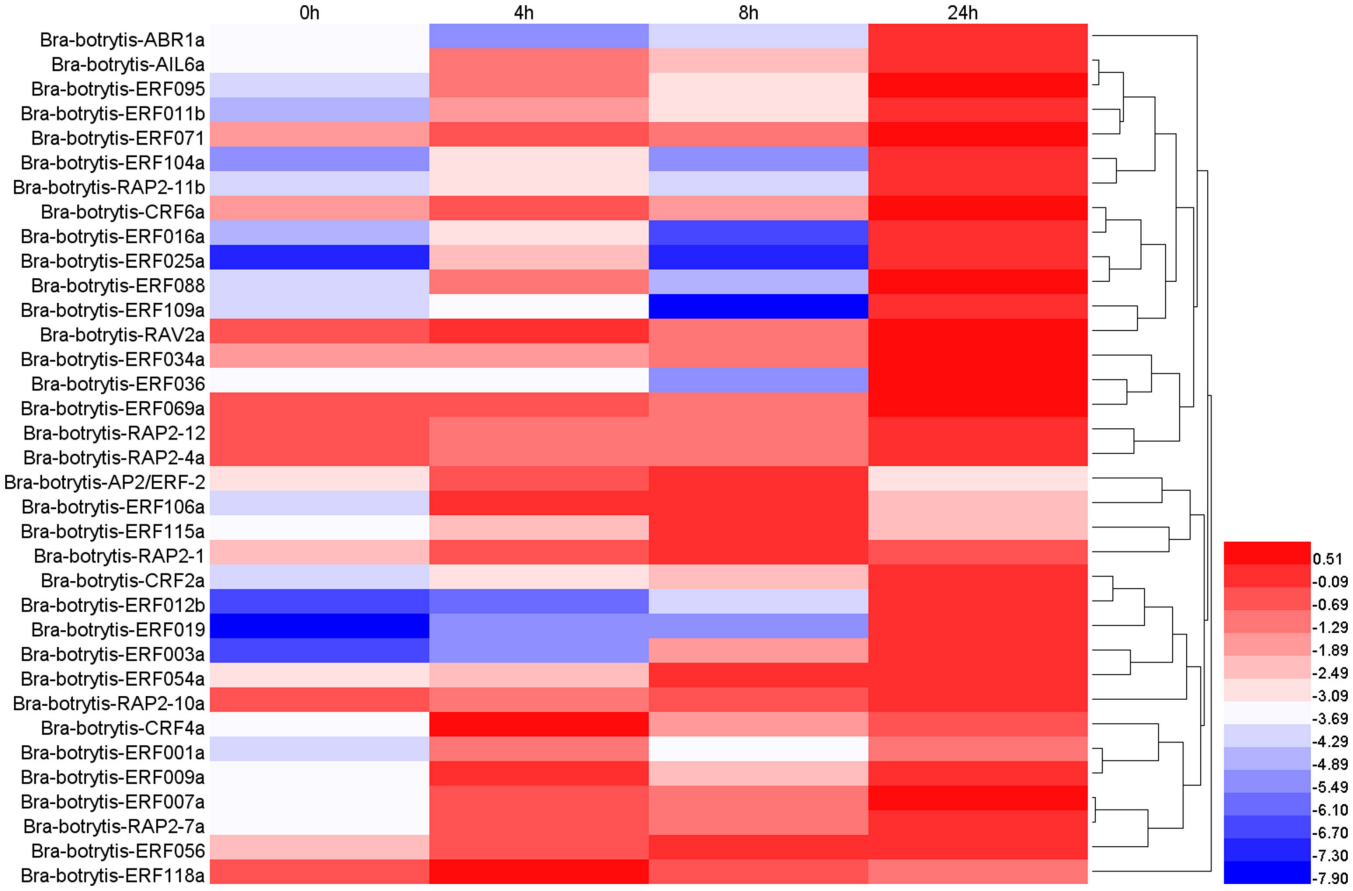
Expression profiles of 35 putative cauliflower AP2/ERF transcription factors at 0 (control), 4, 8, and 24 h after salt treatment. The heat map is constructed based on the Log_2_ (relative expression level of each transcription factor) detected by qRT-PCR. The color scale represents the Log_2_ (relative expression level of each transcription factor) with blue denoting low expression and red denoting high expression.

According to the phylogeny and conserved motif analysis, 35 AP2/ERF TFs were randomly selected and subjected to expression profiling analysis under salt stress. At least two AP2/ERF TFs were selected from each group. All the AP2/ERF TFs mainly exhibited five significantly differential expression patterns (Table S3, Figures 6, **8A–E**). (i) *Bra-botrytis-ERF054a, Bra-botrytis-ERF056, Bra-botrytis-ERF003a*, and *Bra-botrytis-CRF2a* showed fast responses to salt stress. The expression levels of all these TFs increased quickly under high-salt treatment (4 h) and increased continuously under salt stress at 8 and 24 h (**Figure 8B**). (ii) A large proportion of TFs, which included *Bra-botrytis-CRF4a, Bra-botrytis-ERF007a, Bra-botrytis-ERF011b, Bra-botrytis-ERF071, Bra-botrytis-RAP2-7a, Bra-botrytis-AIL6a, Bra-botrytis-CRF6a, Bra-botrytis-ERF001a, Bra-botrytis-ERF009a, Bra-botrytis-ERF025a, Bra-botrytis-ERF088, Bra-botrytis-ERF095*, and *Bra-botrytis-RAV2a* showed rapid responses to salt stress, although their expression levels irregularly increased (**Figure 8A**). (iii) *Bra-botrytis-ABR1a, Bra-botrytis-ERF012b, Bra-botrytis-ERF016a, Bra-botrytis-ERF019, Bra-botrytis-ERF034a, Bra-botrytis-ERF036, Bra-botrytis-ERF069a, Bra-botrytis-ERF104a, Bra-botrytis-ERF109a*, and *Bra-botrytis-RAP2-11b* were also expressed in response to salt stress, although they exhibited delayed response. Compared with the controls (0 h), the expression levels of these TFs did not show considerable changes under salt stress at 4 and 8 h but significantly increased 24 h after salt stress (**Figure 8C**). (iv) Five TFs (*Bra-botrytis-AP2/ERF-2, Bra-botrytis-ERF106a, Bra-botrytis-ERF115a*, and *Bra-botrytis-RAP2-1*) exhibited increased expression at the early stage of salt tress (4 and 8 h). Under prolonged salt treatments, their expression levels decreased (**Figure 8D**). (v) In contrast to (iii), the transcript expression levels of *Bra-botrytis-RAP2-12, Bra-botrytis-RAP2-10a*, and *Bra-botrytis-RAP2-4a* were inhibited in the initial phase of salt stress, but increased with the extension of salt treatments (**Figure 8E**).

### Expression profiling of AP2/ERF TFs under drought stress

**Figure 7:**
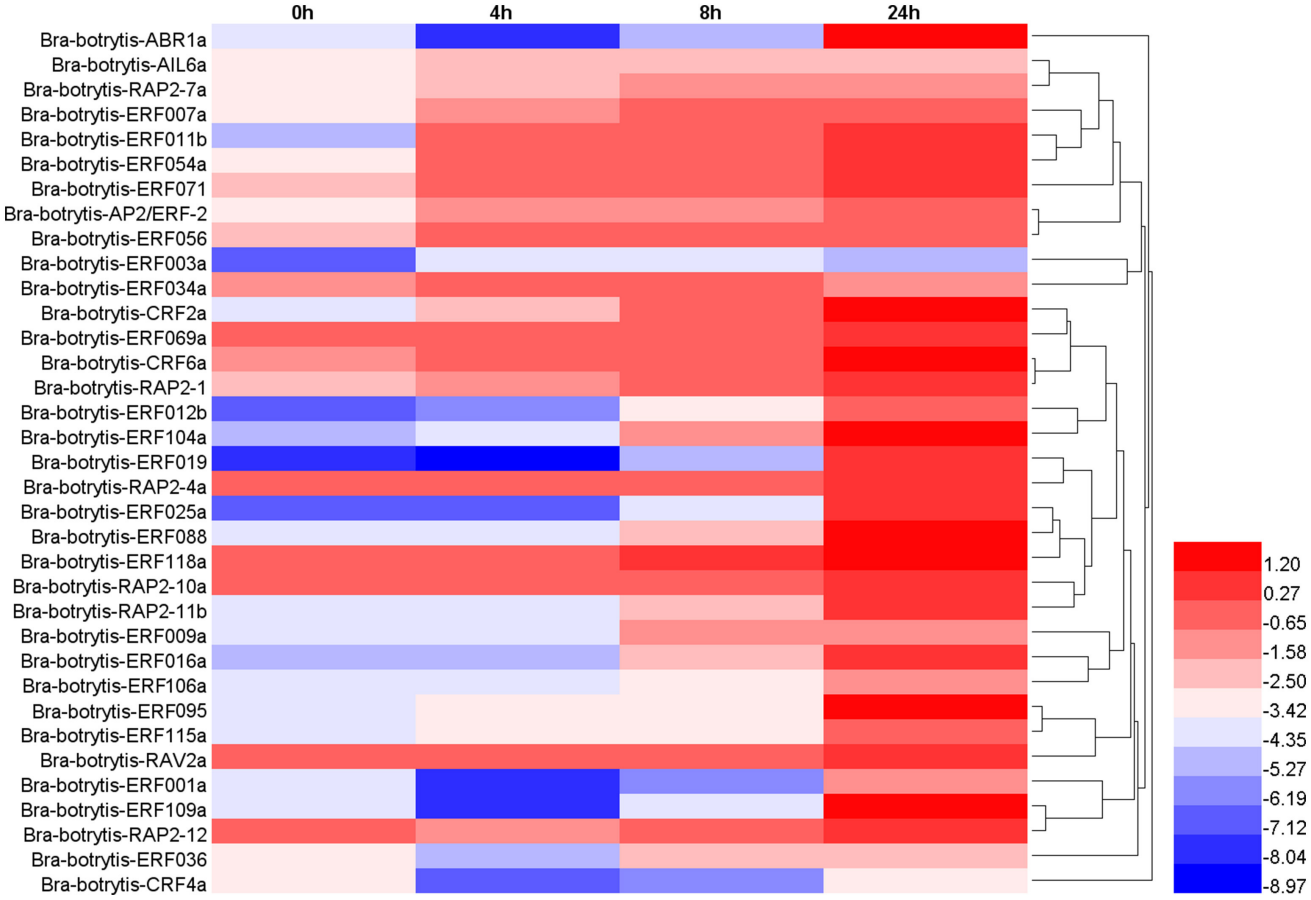
Expression profiles of 35 putative cauliflower AP2/ERF transcription factors at 0 (control), 4, 8, and 24 h after drought treatment. The heat map is constructed based on the Log_2_ (relative expression level of each transcription factor) detected by qRT-PCR. The color scale represents the Log2 (relative expression level of each transcription factor) with blue denoting low expression and red denoting high expression.

**Figure 8:**
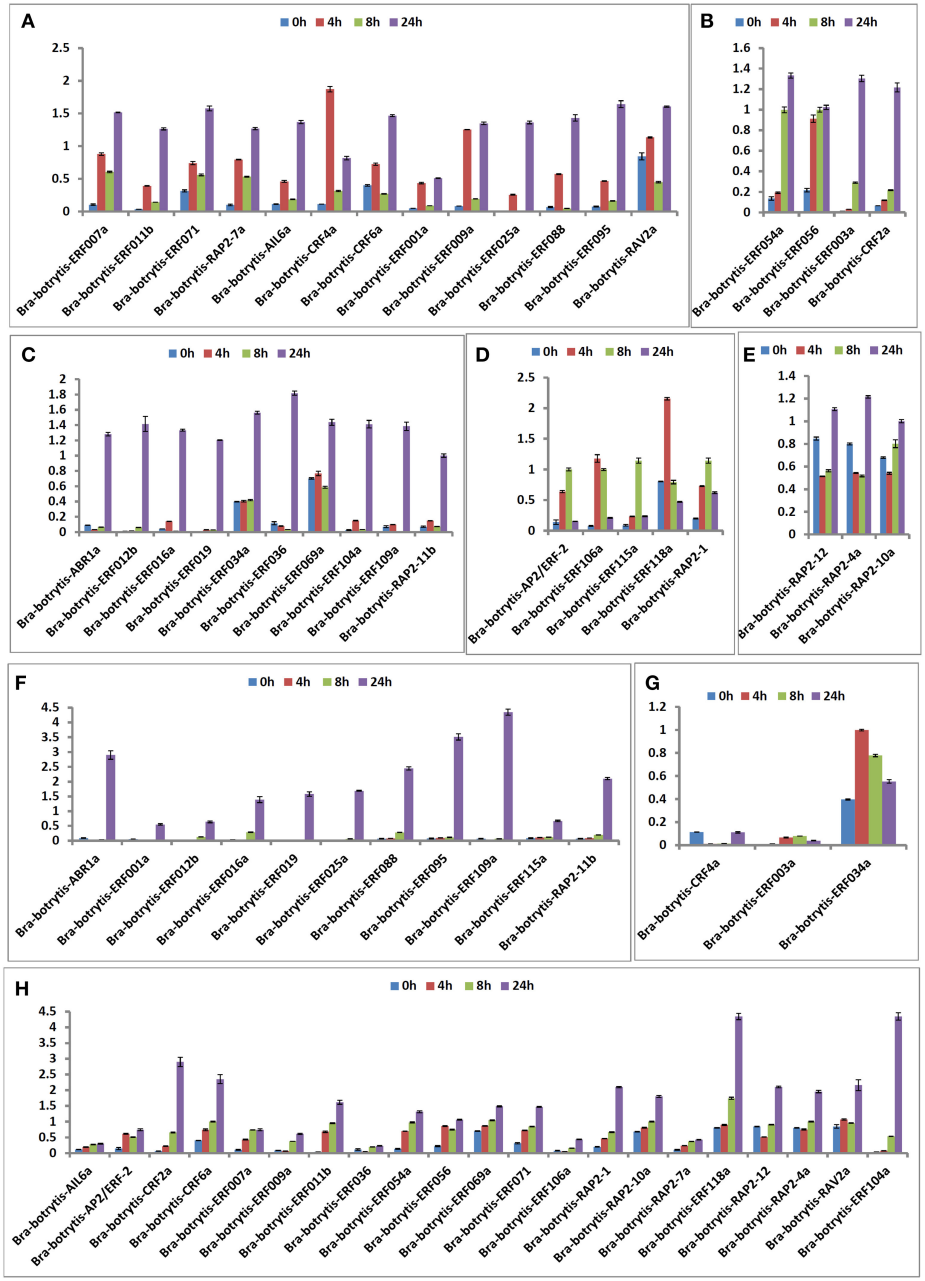
Expression trends of 35 putative cauliflower AP2/ERF transcription factors at 0 (control), 4, 8, and 24 h after salt stress **(A–E)** and drought stress **(F–H)**, respectively.

The expression profiles of the 35 selected AP2/ERF TFs were also explored under drought stress. These TFs mainly exhibited two different expression patterns (Table S4, Figures 7, 8F–H), which differed from their expression patterns under salt stress. In brief, *Bra-botrytis-ABR1a, Bra-botrytis-ERF001a, Bra-botrytis-ERF012b, Bra-botrytis-ERF016a, Bra-botrytis-ERF019, Bra-botrytis-ERF025a, Bra-botrytis-ERF088, Bra-botrytis-ERF095, Bra-botrytis-ERF109a, Bra-botrytis-ERF115a*, and *Bra-botrytis-RAP2-11b* exhibited delayed responses under drought stress. The TFs, such as *Bra-botrytis-AP2/ERF-2, Bra-botrytis-CRF2a, Bra-botrytis-CRF6a, Bra-botrytis-ERF007a, Bra-botrytis-ERF009a, Bra-botrytis-ERF011b, Bra-botrytis-ERF036, Bra-botrytis-ERF054a, Bra-botrytis-ERF056, Bra-botrytis-ERF069a, Bra-botrytis-ERF071, Bra-botrytis-ERF106a, Bra-botrytis-RAP2-1, Bra-botrytis-RAP2-10a*, and *Bra-botrytis-RAP2-7a*, were sensitive to drought stress. The expression levels of these TFs rapidly increased under drought stress. By contrast, two TFs (*Bra-botrytis-CRF4a* and *Bra-botrytis-ERF003a*) did not significantly change their expression levels under drought stress (Table S4, Figures 7, 8F–H).

### Ectopic overexpression of *Bra-botrytis-ERF056*

**Figure 9:**
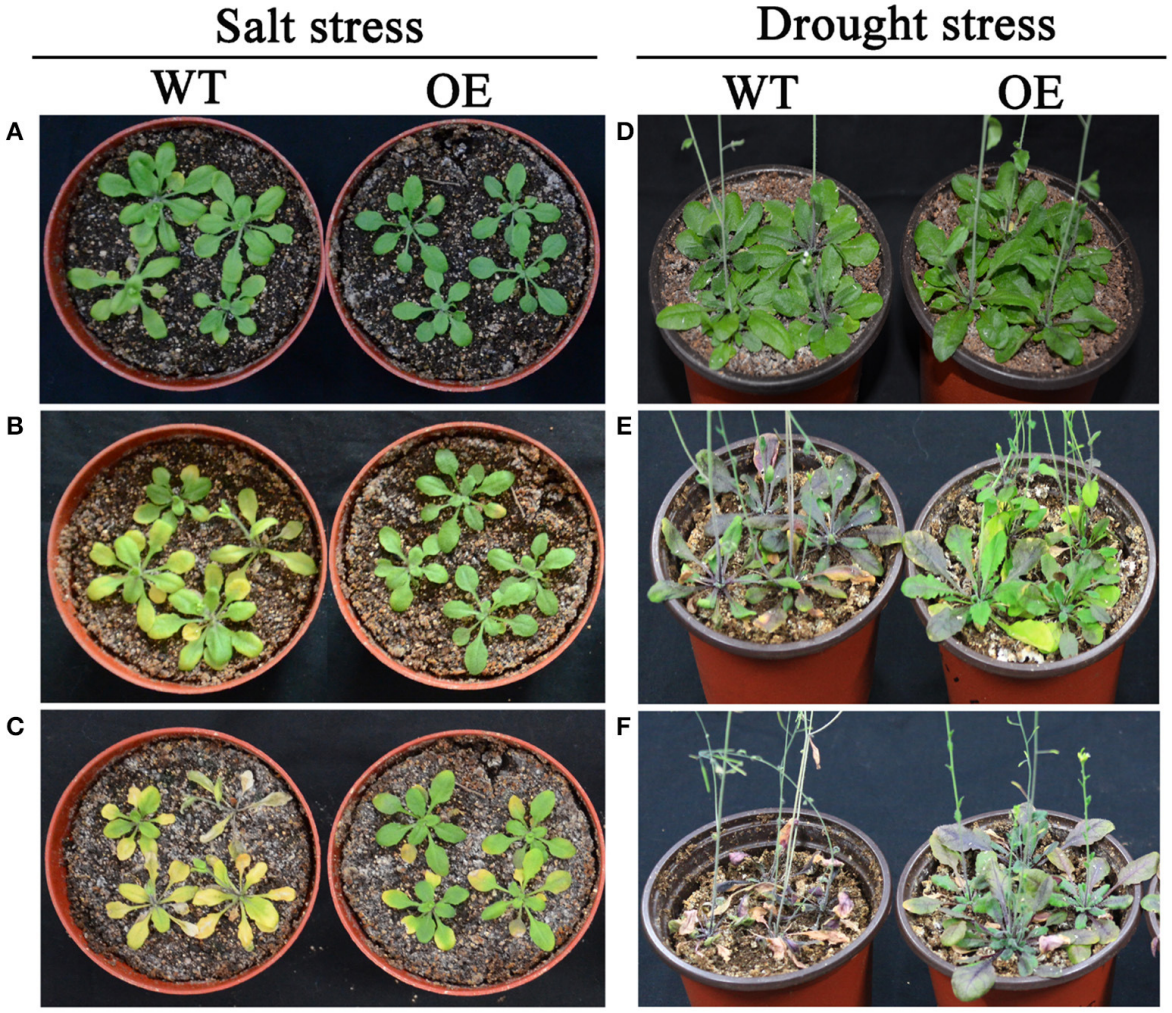
Phenotypes of overexpression *Bra-botrytis-ERF056* transgenic plants under salt and drought stresses. **(A–C)** Indicated the phenotypes of overexpression *Bra-botrytis-ERF056* transgenic plants and wide type controls at 0 day **(A)**, 5 day **(B)**, and 11 day **(C)** after salt tress by 200 mM NaCl treatment. **(D–F)** Indicated the phenotypes of overexpression *Bra-botrytis-ERF056* transgenic plants and wide type controls at 0 day **(D)**, 15 day **(E)**, and 20 day **(F)** without irrigating water. WT showed wide type Arabidopsis plants. OE indicated overexpression *Bra-botrytis-ERF056* transgenic plants.

According to expression profiles of the 35 selected AP2/ERF TFs under salt and drought stresses, three TFs (*Bra-botrytis-ERF054a, Bra-botrytis-ERF056*, and *Bra-botrytis-CRF2a*) demonstrated rapid positive responses to both stresses. To further elucidate the roles of these TFs in response to salt and drought stresses, the function of *Bra-botrytis-ERF056* was explored. The results indicated that transgenic Arabidopsis plants with ectopic overexpression of *Bra-botrytis-ERF056* did not show phenotypic differences from the wide type under the normal growth condition. Under salt treatment, *Bra-botrytis-ERF056* overexpression transgenic plants and wide type controls were irrigated with 200 mM NaCl. After 5 days, the transgenic plants exhibited higher tolerance than those of the wide type controls. At the 11^th^ day after the salt treatment, the wide type plants were almost withered, whereas the transgenic plants were survived and showed more green leaves (Figures 9A–C). Similarly, under drought treatment, after 15 days without irrigating water, the *Bra-botrytis-ERF056* overexpression transgenic plants exhibited higher tolerance than those of the wide type controls. At the 20^th^ days after the drought treatment, the wide type Arabidopsis plants were died, whereas the transgenic plants were survived (Figures 9D–F).

## Discussion

AP2/ERF TFs are almost plant specific (Rashid et al., 2012; Licausi et al., 2013). Thus, identifying AP2/ERF TFs can considerably improve our understanding about the evolution and function of these TFs in various plant species. With the development of next-generation sequencing technology and the release of the genome data of more plant species, the AP2/ERF superfamily has been explored deeply in various plant species. Currently, the AP2/ERF TFs in *Arabidopsis* (Nakano et al., 2006), rice (Rashid et al., 2012), grapevine (Licausi et al., 2010), poplar (Zhuang et al., 2008), Chinese cabbage (Song et al., 2013), Cabbage (Thamilarasan et al., 2014), peach (Zhang et al., 2012), castor bean (Xu et al., 2013), foxtail millet (Lata et al., 2014), switchgrass (Wuddineh et al., 2015), *Musa acuminate, Musa balbisiana* (Lakhwani et al., 2016), and *Brachypodium distachyon* (Chen et al., 2016) have been successfully identified and investigated. Comprehensive data analysis confirmed that more than 100 AP2/ERF TFs have been confirmed in these plants, and 291 and 318 AP2/ERFs TFs were detected in Chinese cabbage and *M. balbisiana*, respectively. To date these TFs represent the highest number of TFs included in the AP2/ERF superfamily of dicots and monocots (Song et al., 2013; Lakhwani et al., 2016). In the present study, 171 AP2/ERF TFs were identified in cauliflower. This number is close to the number of AP2/ERF TFs in foxtail millet (171) (Lata et al., 2014) and rice (170) (Rashid et al., 2012). However, the number of AP2/ERF TFs in cauliflower is lower than that in Chinese cabbage (291) and cabbage (226) even though these three plants belong to the same genus. Further phylogenetic tree analysis of AP2/ERF TFs from cauliflower, Chinese cabbage and cabbage indicated that the number of TFs from AP2 family and DERB subfamily in cauliflower were significantly less than that in Chinese cabbage and cabbage. Compared to cauliflower, a lot of TFs in these two groups, especially in AP2 family, occurred gene duplication event in Chinese cabbage and cabbage. Reversely, more TFs from ERF subfamily were identified in cauliflower than those in cabbage. Compared to cabbage, a few ERF TFs experienced duplication in cauliflower. The number of TFs in RAV and Soloist families was no significantly different in these three plants (Figure S2). These results indicated that the different number of AP2/ERF TFs in cauliflower from its two closely related species mainly resulted by the contractions of the AP2 and DREB TFs, and the expansions of some ERF TFs. Previous report confirmed that during evolution, Brassica genus including cauliflower, Chinese cabbage and cabbage underwent genome triplication (Cheng et al., 2016). The genome triplication event may be directly involved in the evolution of AP2/ERF TFs in different plants. Alternatively, the absence of whole genome data of cauliflower is an unavoidable factor that results in the omission of some AP2/ERF TFs, although high-throughput transcriptome data from different organs of cauliflower were used. Nevertheless, the present study offered significant clues to elucidate further the evolution and function of AP2/ERF TFs in cauliflower.

In general, the domains or amino acid motifs of TFs are frequently involved in DNA- binding, nuclear localization, protein- protein interaction, and transcriptional activity (Nakano et al., 2006). TFs with similar conserved domains or amino acid motifs are likely to have similar functions. Consequently, the conserved motifs in cauliflower AP2/ERF TFs were further analyzed. A total of 25 motifs were identified in the 171 cauliflower AP2/ERF TFs. Except motif-1, motif-2, motif-3, and motif-4, which were in the AP2 domain regions and existed in almost all AP2/ERF TFs, most motifs showed group- or subgroup- specific distributions (Figures 2–5). Motif-5 and motif-9 were specifically present in the B3 domain, and the TFs contained these two motifs were all classified into group VII and were member of the RAV family. Consisted with TFs of this family in other plants such as *Arabidopsis* and switchgrass, the core sequence of the B3 domain (RLFGV) was also detected in the RAV TFs of cauliflower (Ikeda and Ohme-Takagi, 2009). Some of the RAV TFs, such as RAV1 and RAV2, were confirmed as transcriptional repressors in plants (Hu et al., 2004; Mittal et al., 2015). Motif-6, motif-17, motif-18, and motif-23 were group VIII- specific, in which motif-17 and motif-23 containing the CRF (cytokinin responsive factor) domain were only present in the CRF TFs of this group (Rashotte et al., 2006). In motif-17, a more conserved sequence (SP(T/V)SVL) was identified, which was predicted to function as a putative MAP kinase phosphorylation site (Nakano et al., 2006). In *Arabidopsis* TFs containing the CRF domain exhibited responses to cytokinin (Rashotte et al., 2006). The homologous CRF TFs were also detected in other plants such as switchgrass and moso bamboo (Wuddineh et al., 2015; Wu et al., 2015). Inside the two AP2 domains, motif-7 was confirmed to be present only in the N-terminus of the AP2 family. Alignment result analysis indicated that the amino acid sequences of this motif were also detected in the AP2 TFs of various plant species, but the role of this motif was unknown. Motif-10 mostly containing conserved D(I/V)QAA sequences was specifically present in groups I and III. The members of these two groups were all from the DREB subfamily. The D(I/V)QAA motif and another motif (LPRP) near the motif-3 are the essential signatures for the TFs from the DREB subfamily to function in response to various stresses (Albrecht et al., 2001; Qu and Zhu, 2006). Conserved motifs such as the ERF-associated amphiphilic repression (EAR) motif and a unique “EDLL” motif that was previously characterized as a transcriptional activation domain in other plants, were also identified in the AP2/ERF TFs in cauliflower (Ohta et al., 2001; Kagale and Rozwadowski, 2011; Tiwari et al., 2012). In addition, few reports have explored the characteristics of other motifs detected only in the present study. These motifs also showed group specificity. For example, motif-11, motif-12, motif-13, motif-14, motif-15, motif-19, motif-20, motif-21, and motif-25 were specific in different groups of the ERF subfamily, implying their important roles for TFs in this subfamily. Species-specific motifs within AP2/ERF TFs were also detected in other plant species (Wuddineh et al., 2015; Lakhwani et al., 2016; Shu et al., 2016). These results indicated that although some domains or motifs of AP2/ERF TFs were highly conserved, newly evolved motifs generated, which may play important roles in the subfunctionalization or new function of AP2/ERF TFs in specific plant species. The function and regulation of these newly evolved motifs in AP2/ERF TFs require further elucidation.

Different from animals, plants must adapt to various biotic and abiotic stresses in their life cycles. In these processes, a few TFs were mobilized to regulate their target genes and made the plants exhibit resistant phenotypes. Among these TFs, some AP2/ERF TFs play important roles in plants to defend themselves against environmental stimuli and improve resistance (Sakuma et al., 2006; Dietz et al., 2010; Mizoi et al., 2012; Shu et al., 2016; Tang et al., 2016). In the present study, to explore the potential roles of the AP2/ERF TFs in response to abiotic stresses in cauliflower, 35 AP2/ERF TFs were selected to conduct expression profiling analysis with salt and drought stresses. Almost all 35 AP2/ERF TFs were activated under these two stresses, whereas most of their expression profiles were different. *Bra-botrytis-ERF003a* continuously increased its expression level under salt stress. However, the expression level of this TF showed no significant change under drought stress. *Bra-botrytis-ERF003a* containing the EAR motif is a homolog of *ERF3* in other plants. *ERF3* as a transcription repressor is involved in various abiotic stresses, leaf senescence, and pathogen defense (Fujimoto et al., 2000; Koyama et al., 2013; Velivelli et al., 2015). *Bra-botrytis-ERF036, Bra-botrytis-ERF069a*, and *Bra-botrytis-ERF104a* exhibited delayed responses to salt stress. The expression levels of these three TFs significantly increased until 8 h under salt stress. Inversely, these TFs rapidly responded to drought stress. The homologous gene of *Bra-botrytis-ERF104a* responded to light stress and pathogenic bacterial infections in *Arabidopsis* (Bethke et al., 2009; Chen et al., 2013; Vogel et al., 2014). Several AP2/ERF TFs, such as *Bra-botrytis-AP2/ERF-2, Bra-botrytis-ERF106a, Bra-botrytis-ERF118a* and *Bra-botrytis-RAP2-1*, were only significantly upregulated at the early stages of salt stress. These AP2/ERF TFs continuously increased their expression levels under drought stress. Similar to *Bra-botrytis-RAP2-1, RAP2.1*, the homolog of *Bra-botrytis-RAP2-1*, was strongly induced by drought and cold stresses in *Arabidopsis*. Overexpression of *RAP2.1* could enhance sensitivity to cold and drought stresses (Fowler and Thomashow, 2002; Dong and Liu, 2010). Homology analysis indicated that *Bra-botrytis-AP2/ERF-2* and four other TFs showed low sequence similarity to reported AP2/ERF TFs. This finding implied that these TFs are specific to cauliflower. Similarly, although *Bra-botrytis-RAP2-12, Bra-botrytis-RAP2-4a*, and *Bra-botrytis-RAP2-10a* showed fast responses to drought stress, the expression of these TFs was inhibited in the early stages of salt stress. *RAP2.12*, the homologous gene of *Bra-botrytis-RAP2-12*, played a vital role in anaerobic response and was involved in the root hydraulics of *Arabidopsis* (Gasch et al., 2016; Paul et al., 2016; Shahzad et al., 2016). *RAP2.4*, the homolog of *Bra-botrytis-RAP2-4a*, was involved in drought stress tolerance in *Arabidopsis* (Lin et al., 2008). This TF also functioned in cold and heat tolerance in papaya tree (Figueroa-Yañez et al., 2016). Other AP2/ERF TFs, such as *Bra-botrytis-CRF4a, Bra-botrytis-CRF6a*, and *Bra-botrytis-ERF001a*, showed different expression profiles under salt and drought stresses. The homologs of some of these AP2/ERF TFs were also involved in various abiotic stresses (Cheng et al., 2013; Zwack et al., 2016a,b). The different expression profiles of these AP2/ERF TFs implied that the functions of these TFs under salt and drought stresses may be different. Meanwhile, the expression profiles of several AP2/ERF TFs were similar under salt and drought stresses. The expression levels of *Bra-botrytis-ERF054a, Bra-botrytis-ERF056*, and *Bra-botrytis-CRF2a* increased under salt and drought stresses. *Bra-botrytis-ABR1a, Bra-botrytis-ERF012b, Bra-botrytis-ERF016a, Bra-botrytis-ERF019, Bra-botrytis-ERF109a*, and *Bra-botrytis-RAP2-11b* all exhibited delayed responses to both stresses. The homologs of these TFs, such as *ABR1* (Pandey et al., 2005), *CRF2* (Jeon et al., 2016), *ERF19* (Kloppholz et al., 2011), *ERF109* (Bahieldin et al., 2016), and *RAP2.11* (Kim et al., 2012) in other plants, were confirmed to respond to abiotic stresses or pathogen infection. The similar expression profiles of these AP2/ERF TFs indicated that they may play similar roles in the defense responses against these two stresses. Consistent with this speculation, in the present study, ectopic overexpression of *Bra-botrytis-ERF056* demonstrated to increase tolerance to both salt and drought stresses (Figure 9). Nevertheless, although a few homologous genes of cauliflower AP2/ERF TFs have been demonstrated to play important roles in tolerance to various biotic and abiotic stresses in other plant species, the functions of AP2/ERF TFs in cauliflower are still largely unknown. These findings provided potential AP2/ERF TF candidates, specifically *Bra-botrytis-ERF056*, to further explore their roles under salt and drought stresses in cauliflower.

In conclusion, 171 AP2/ERF TFs were identified in cauliflower. Clustering and phylogenetic analysis were conducted to divide these TFs into 13 groups. Twenty-five conserved motifs in the 171 AP2/ERF TFs were identified. The AP2/ERF TFs exhibiting specific expression patterns under salt or drought stress were also confirmed. Ectopic overexpression of *Bra-botrytis-ERF056* was confirmed to increase tolerance to both salt and drought treatments. These findings provide new insights into the AP2/ERF TFs in cauliflower and offer candidate AP2/ERF TFs to further elucidate their roles in salt and drought stress tolerance.

## Author contributions

HL performed the experiments, analyzed the data and wrote the manuscript; YW, LL, CL, and ZH performed the experiments; MW, JY, and CC analyzed the data; WS analyzed the data and wrote the manuscript; CW designed the project, analyzed the data and wrote the manuscript.

### Conflict of interest statement

The authors declare that the research was conducted in the absence of any commercial or financial relationships that could be construed as a potential conflict of interest.

## References

[B1] Albrecht V. Ritz O. Linder S. Harter K. Kudla J. (2001). The NAF domain defines a novel protein-protein interaction module conserved in Ca^2+^-regulated kinases. EMBO J. 20, 1051–1063. 10.1093/emboj/20.5.1051 11230129 PMC145464

[B2] Allen M. D. Yamasaki K. Ohme-Takagi M. Tateno M. Suzuki M. (1998). A novel mode of DNA recognition by a beta-sheet revealed by the solution structure of the GCC-box binding domain in complex with DNA. EMBO J. 17, 5484–5496. 10.1093/emboj/17.18.5484 9736626 PMC1170874

[B3] Altschul S. F. Gish W. Miller W. Myers E. W. Lipman D. J. (1990). Basic local alignment search tool. J. Mol. Biol. 215, 403–410. 10.1016/S0022-2836(05)80360-2 2231712

[B4] Aukerman M. J. Sakai H. (2003). Regulation of flowering time and floral organ identity by a microRNA and its APETALA2-like target genes. Plant Cell 15, 2730–2741. 10.1105/tpc.016238 14555699 PMC280575

[B5] Bahieldin A. Atef A. Edris S. Gadalla N. O. Ali H. M. Hassan S. M.. (2016). Ethylene responsive transcription factor *ERF109* retards PCD and improves salt tolerance in plant. BMC Plant Biol. 16:216. 10.1186/s12870-016-0908-z 27716054 PMC5053207

[B6] Bailey T. L. Boden M. Buske F. A. Frith M. Grant C. E. Clementi L.. (2009). MEME SUITE: tools for motif discovery and searching. Nucleic Acids Res. 37, W202–W208. 10.1093/nar/gkp335 19458158 PMC2703892

[B7] Bethke G. Unthan T. Uhrig J. F. Pöschl Y. Gust A. A. Scheel D.. (2009). Flg22 regulates the release of an ethylene response factor substrate from MAP kinase 6 in Arabidopsis thaliana via ethylene signaling. Proc. Natl. Acad. Sci. U.S.A. 106, 8067–8072. 10.1073/pnas.0810206106 19416906 PMC2683104

[B8] Bouaziz D. Pirrello J. Charfeddine M. Hammami A. Jbir R. Dhieb A.. (2013). Overexpression of *StDREB1* transcription factor increases tolerance to salt in transgenic potato plants. Mol. Biotechnol. 54, 803–817. 10.1007/s12033-012-9628-2 23250722

[B9] Chen L. Han J. Deng X. Tan S. Li L. Li L.. (2016). Expansion and stress responses of AP2/EREBP superfamily in *Brachypodium distachyon*. Sci. Rep. 6:21623. 10.1038/srep21623 26869021 PMC4751504

[B10] Chen L. Zhang L. Li D. Wang F. Yu D. (2013). *WRKY8* transcription factor functions in the TMV-cg defense response by mediating both abscisic acid and ethylene signaling in Arabidopsis. Proc. Natl. Acad. Sci. U.S.A. 110, E1963–E1971. 10.1073/pnas.1221347110 23650359 PMC3666684

[B11] Cheng F. Sun R. Hou X. Zheng H. Zhang F. Zhang Y.. (2016). Subgenome parallel selection is associated with morphotype diversification and convergent crop domestication in *Brassica rapa* and *Brassica oleracea*. Nat. Genet. 48, 1218–1224. 10.1038/ng.3634 27526322

[B12] Cheng M. C. Liao P. M. Kuo W. W. Lin T. P. (2013). The Arabidopsis *ETHYLENE RESPONSE FACTOR1* regulates abiotic stress-responsive gene expression by binding to different cis-acting elements in response to different stress signals. Plant Physiol. 162, 1566–1582. 10.1104/pp.113.221911 23719892 PMC3707555

[B13] Clough S. J. Bent A. F. (1998). Floral dip: a simplified method for Agrobacterium-mediated transformation of *Arabidopsis thaliana*. Plant J. 16, 735–743. 10.1046/j.1365-313x.1998.00343.x 10069079

[B14] Dietz K. J. Vogel M. O. Viehhauser A. (2010). AP2/EREBP transcription factors are part of gene regulatory networks and integrate metabolic, hormonal and environmental signals in stress acclimation and retrograde signalling. Protoplasma 245, 3–14. 10.1007/s00709-010-0142-8 20411284

[B15] Dong C. J. Liu J. Y. (2010). The Arabidopsis EAR-motif-containing protein RAP2.1 functions as an active transcriptional repressor to keep stress responses under tight control. BMC Plant Biol. 10:47. 10.1186/1471-2229-10-47 20230648 PMC2848764

[B16] Dong L. Cheng Y. Wu J. Cheng Q. Li W. Fan S.. (2015). Overexpression of *GmERF5*, a new member of the soybean EAR motif-containing ERF transcription factor, enhances resistance to *Phytophthora sojae* in soybean. J. Exp. Bot. 66, 2635–2647. 10.1093/jxb/erv078 25779701

[B17] Fang Z. Zhang X. Gao J. Wang P. Xu X. Liu Z. (2015). A buckwheat (*Fagopyrum esculentum*) DRE-Binding transcription factor gene, *FeDREB1*, enhances freezing and drought tolerance of transgenic Arabidopsis. Plant Mol. Biol. Rep. 33, 1510 10.1007/s11105-015-0851-4

[B18] Figueroa-Yañez L. Pereira-Santana A. Arroyo-Herrera A. Rodriguez-Corona U. Sanchez-Teyer F. Espadas-Alcocer J.. (2016). *RAP2.4a* is transported through the phloem to regulate cold and heat tolerance in papaya Tree (*Carica papaya* cv. Maradol): implications for protection against abiotic stress. PLoS ONE 11:e0165030. 10.1371/journal.pone.0165030 27764197 PMC5072549

[B19] Finn R. D. Clements J. Arndt W. Miller B. L. Wheeler T. J. Schreiber F.. (2015). HMMER web server: 2015 update. Nucleic Acids Res. 43, W30–W38. 10.1093/nar/gkv397 25943547 PMC4489315

[B20] Fowler S. Thomashow M. F. (2002). Arabidopsis transcriptome profiling indicates that multiple regulatory pathways are activated during cold acclimation in addition to the CBF cold response pathway. Plant Cell 14, 1675–1690. 10.1105/tpc.003483 12172015 PMC151458

[B21] Fujimoto S. Y. Ohta M. Usui A. Shinshi H. Ohme-Takagi M. (2000). Arabidopsis ethylene-responsive element binding factors act as transcriptional activators or repressors of GCC box-mediated gene expression. Plant Cell 12, 393–404. 10.1105/tpc.12.3.393 10715325 PMC139839

[B22] Fujita Y. Fujita M. Shinozaki K. Yamaguchi-Shinozaki K. (2011). ABA-mediated transcriptional regulation in response to osmotic stress in plants. J. Plant Res. 124, 509–525. 10.1007/s10265-011-0412-3 21416314

[B23] Gasch P. Fundinger M. Müller J. T. Lee T. Bailey-Serres J. Mustroph A. (2016). Redundant ERF-VII transcription factors bind to an evolutionarily conserved cis-Motif to regulate hypoxia-responsive gene expression in Arabidopsis. Plant Cell 28, 160–180. 10.1105/tpc.15.00866 26668304 PMC4746684

[B24] Hong J. P. Kim W. T. (2005). Isolation and functional characterization of the *Ca-DREBLP1* gene encoding a dehydration-responsive element binding-factor-like protein 1 in hot pepper (*Capsicum annuum* L. cv. Pukang). Planta 220, 875–888. 10.1007/s00425-004-1412-5 15538622

[B25] Horstman A. Willemsen V. Boutilier K. Heidstra R. (2014). AINTEGUMENTA-LIKE proteins: hubs in a plethora of networks. Trends Plant Sci. 19, 146–157. 10.1016/j.tplants.2013.10.010 24280109

[B26] Hu Y. X. Wang Y. X. Liu X. F. Li J. Y. (2004). Arabidopsis *RAV1* is down-regulated by brassinosteroid and may act as a negative regulator during plant development. Cell Res. 14, 8–15. 10.1038/sj.cr.7290197 15040885

[B27] Ikeda M. Ohme-Takagi M. (2009). A novel group of transcriptional repressors in Arabidopsis. Plant Cell Physiol. 50, 970–975. 10.1093/pcp/pcp048 19324928

[B28] Ito Y. Katsura K. Maruyama K. Taji T. Kobayashi M. Seki M.. (2006). Functional analysis of rice DREB1/CBF-type transcription factors involved in cold-responsive gene expression in transgenic rice. Plant Cell Physiol. 47, 141–153. 10.1093/pcp/pci230 16284406

[B29] Jeon J. Cho C. Lee M. R. Van Binh N. Kim J. (2016). *CYTOKININ RESPONSE FACTOR2 (CRF2)* and *CRF3* regulate lateral root development in response to cold stress in Arabidopsis. Plant Cell 28, 1828–1843. 10.1105/tpc.15.00909 27432872 PMC5006697

[B30] Jofuku K. D. Omidyar P. K. Gee Z. Okamuro J. K. (2005). Control of seed mass and seed yield by the floral homeotic gene *APETALA2*. Proc. Natl. Acad. Sci. U.S.A. 102, 3117–3122. 10.1073/pnas.0409893102 15708974 PMC549499

[B31] Joung J. K. Sander J. D. (2013). TALENs: a widely applicable technology for targeted genome editing. Nat. Rev. Mol. Cell Biol. 14, 49–55. 10.1038/nrm3486 23169466 PMC3547402

[B32] Kagale S. Rozwadowski K. (2011). EAR motif-mediated transcriptional repression in plants: an underlying mechanism for epigenetic regulation of gene expression. Epigenetics 6, 141–146. 10.4161/epi.6.2.13627 20935498 PMC3278782

[B33] Kim M. J. Ruzicka D. Shin R. Schachtman D. P. (2012). The Arabidopsis AP2/ERF transcription factor *RAP2.11* modulates plant response to low-potassium conditions. Mol. Plant 5, 1042–1057. 10.1093/mp/sss003 22406475

[B34] Kloppholz S. Kuhn H. Requena N. (2011). A secreted fungal effector of Glomus intraradices promotes symbiotic biotrophy. Curr. Biol. 21, 1204–1209. 10.1016/j.cub.2011.06.044 21757354

[B35] Koyama T. Nii H. Mitsuda N. Ohta M. Kitajima S. Ohme-Takagi M.. (2013). A regulatory cascade involving class II ETHYLENE RESPONSE FACTOR transcriptional repressors operates in the progression of leaf senescence. Plant Physiol. 162, 991–1005. 10.1104/pp.113.218115 23629833 PMC3668086

[B36] Kuluev B. Avalbaev A. Nurgaleeva E. Knyazev A. Nikonorov Y. Chemeris A. (2015). Role of AINTEGUMENTA-like gene *NtANTL* in the regulation of tobacco organ growth. J. Plant Physiol. 189, 11–23. 10.1016/j.jplph.2015.08.009 26479044

[B37] Lakhwani D. Pandey A. Dhar Y. V. Bag S. K. Trivedi P. K. Asif M. H. (2016). Genome-wide analysis of the AP2/ERF family in Musa species reveals divergence and neofunctionalisation during evolution. Sci. Rep. 6:18878. 10.1038/srep18878 26733055 PMC4702079

[B38] Lata C. Mishra A. K. Muthamilarasan M. Bonthala V. S. Khan Y. Prasad M. (2014). Genome-wide investigation and expression profiling of AP2/ERF transcription factor superfamily in foxtail millet (*Setariaitalica* L.). PLoS ONE 9:e113092. 10.1371/journal.pone.0113092 25409524 PMC4237383

[B39] Li A. Zhou Y. Jin C. Song W. Chen C. Wang C. (2013). *LaAP2L1*, a heterosis-associated AP2/EREBP transcription factor of *Larix*, increases organ size and final biomass by affecting cell proliferation in Arabidopsis. Plant Cell Physiol. 54, 1822–1836. 10.1093/pcp/pct124 24009335

[B40] Licausi F. Giorgi F. M. Zenoni S. Osti F. Pezzotti M. Perata P. (2010). Genomic and transcriptomic analysis of the AP2/ERF superfamily in *Vitis vinifera*. BMC Genomics 11:719. 10.1186/1471-2164-11-719 21171999 PMC3022922

[B41] Licausi F. Ohme-Takagi M. Perata P. (2013). APETALA2/Ethylene responsive factor (AP2/ERF) transcription factors: mediators of stress responses and developmental programs. New Phytol. 199, 639–649. 10.1111/nph.12291 24010138

[B42] Lin R. C. Park H. J. Wang H. Y. (2008). Role of Arabidopsis *RAP2.4* in regulating light-and ethylene-mediated developmental processes and drought stress tolerance. Mol. Plant 1, 42–57. 10.1093/mp/ssm004 20031913

[B43] Mantiri F. R. Kurdyukov S. Lohar D. P. Sharopova N. Saeed N. A. Wang X. D.. (2008). The transcription factor *MtSERF1* of the ERF subfamily identified by transcriptional profiling is required for somatic embryogenesis induced by auxin plus cytokinin in *Medicago truncatula*. Plant Physiol. 146, 1622–1636. 10.1104/pp.107.110379 18235037 PMC2287338

[B44] Mittal A. Gampala S. S. Ritchie G. L. Payton P. Burke J. J. Rock C. D. (2014). Related to ABA-Insensitive3 (ABI3)/Viviparous1 and AtABI5 transcription factor coexpression in cotton enhances drought stress adaptation. Plant Biotechnol. J. 12, 578–589. 10.1111/pbi.12162 24483851 PMC4043863

[B45] Mittal A. Jiang Y. Ritchie G. L. Burke J. J. Rock C. D. (2015). *AtRAV1* and *AtRAV2* overexpression in cotton increases fiber length differentially under drought stress and delays flowering. Plant Sci. 241, 78–95. 10.1016/j.plantsci.2015.09.013 26706061

[B46] Mizoi J. Shinozaki K. Yamaguchi-Shinozaki K. (2012). AP2/ERF family transcription factors in plant abiotic stress responses. Biochim. Biophys. Acta 1819, 86–96. 10.1016/j.bbagrm.2011.08.004 21867785

[B47] Moose S. P. Sisco P. H. (1996). *Glossy15*, an APETALA2-like gene from maize that regulates leaf epidermal cell identity. Genes Dev. 10, 3018–3027. 10.1101/gad.10.23.3018 8957002

[B48] Nakano T. Suzuki K. Fujimura T. Shinshi H. (2006). Genome-wideanalysis of the ERF gene family in Arabidopsis and rice. Plant Physiol. 140, 411–432. 10.1104/pp.105.073783 16407444 PMC1361313

[B49] Ohme-Takagi M. Shinshi H. (1995). Ethylene-inducible DNA binding proteins that interact with an ethylene-responsive element. Plant Cell 7, 173–182. 10.1105/tpc.7.2.173 7756828 PMC160773

[B50] Ohta M. Matsui K. Hiratsu K. Shinshi H. Ohme-Takagi M. (2001). Repression domains of class II ERF transcriptional repressors share an essential motif for active repression. Plant Cell 13, 1959–1968. 10.1105/tpc.13.8.1959 11487705 PMC139139

[B51] Oñate-Sánchez L. Singh K. B. (2002). Identification of Arabidopsis ethylene-responsive element binding factors with distinct induction kinetics after pathogen infection. Plant Physiol. 128, 1313–1322. 10.1104/pp.010862 11950980 PMC154259

[B52] Pandey G. K. Grant J. J. Cheong Y. H. Kim B. G. Li L. Luan S. (2005). *ABR1*, an APETALA2-domain transcription factor that functions as a repressor of ABA response in Arabidopsis. Plant Physiol. 139, 1185–1193. 10.1104/pp.105.066324 16227468 PMC1283757

[B53] Paul M. V. Iyer S. Amerhauser C. Lehmann M. van Dongen J. T. Geigenberger P. (2016). Oxygen Sensing via the ethylene response transcription factor *RAP2.12* affects plant metabolism and performance under both normoxia and hypoxia. Plant Physiol. 172, 141–153. 10.1104/pp.16.00460 27372243 PMC5074624

[B54] Qin F. Kakimoto M. Sakuma Y. Maruyama K. Osakabe Y. Tran L. S.. (2007). Regulation and functional analysis of *ZmDREB2A* in response to drought and heat stresses in *Zea mays* L. Plant J. 50, 54–69. 10.1111/j.1365-313X.2007.03034.x 17346263

[B55] Qu L. J. Zhu Y. X. (2006). Transcription factor families in Arabidopsis: major progress and outstanding issues for future research. Curr. Opin. Plant Biol. 9, 544–549. 10.1016/j.pbi.2006.07.005 16877030

[B56] Rashid M. Guangyuan H. Guangxiao Y. Hussain J. Xu Y. (2012). AP2/ERF Transcription factor in rice: genome-wide canvas and syntenic relationships between monocots and eudicots. Evol. Bioinform. Online 8, 321–355. 10.4137/EBO.S9369 22807623 PMC3396566

[B57] Rashotte A. M. Mason M. G. Hutchison C. E. Ferreira F. J. Schaller G. E. Kieber J. J. (2006). A subset of Arabidopsis AP2 transcription factors mediates cytokinin responses in concert with a two-component pathway. Proc. Natl. Acad. Sci. U.S.A. 103, 11081–11085. 10.1073/pnas.0602038103 16832061 PMC1544176

[B58] Sakuma Y. Maruyama K. Osakabe Y. Qin F. Seki M. Shinozaki K.. (2006). Functional analysis of an Arabidopsis transcription factor, *DREB2A*, involved in drought-responsive gene expression. Plant Cell 18, 1292–1309. 10.1105/tpc.105.035881 16617101 PMC1456870

[B59] Seo Y. J. Park J. B. Cho Y. J. Jung C. Seo H. S. Park S. K.. (2010). Overexpression of the ethylene-responsive factor gene *BrERF4* from Brassica rapa increases tolerance to salt and drought in Arabidopsis plants. Mol. Cells 30, 271–277. 10.1007/s10059-010-0114-z 20803085

[B60] Shahzad Z. Canut M. Tournaire-Roux C. Martinière A. Boursiac Y. Loudet O.. (2016). A potassium-dependent oxygen sensing pathway regulates plant root hydraulics. Cell 167, 87–98. 10.1016/j.cell.2016.08.068 27641502

[B61] Shan Q. Wang Y. Li J. Gao C. (2014). Genome editing in rice and wheat using the CRISPR/Cas system. Nat. Protoc. 9, 2395–2410. 10.1038/nprot.2014.157 25232936

[B62] Shu Y. Liu Y. Zhang J. Song L. Guo C. (2016). Genome-wide analysis of the AP2/ERF superfamily genes and their responses to abiotic stress in *Medicago truncatula*. Front. Plant Sci. 6:1247. 10.3389/fpls.2015.01247 26834762 PMC4717309

[B63] Song X. Li Y. Hou X. (2013). Genome-wide analysis of the AP2/ERF transcription factor superfamily in Chinese cabbage (*Brassica rapa* ssp. pekinensis). BMC Genomics 14:573. 10.1186/1471-2164-14-573 23972083 PMC3765354

[B64] Sun S. Yu J. P. Chen F. Zhao T. J. Fang X. H. Li Y. Q.. (2008). TINY, a dehydration-responsive element (DRE)-binding protein-like transcription factor connecting the DRE- and ethylene-responsive element-mediated signaling pathways in Arabidopsis. J. Biol. Chem. 283, 6261–6271. 10.1074/jbc.M706800200 18089556

[B65] Tamura K. Stecher G. Peterson D. Filipski A. Kumar S. (2013). MEGA6: molecular evolutionary genetics analysis version 6.0. Mol. Biol. Evol. 30, 2725–2729. 10.1093/molbev/mst197 24132122 PMC3840312

[B66] Tang Y. Qin S. Guo Y. Chen Y. Wu P. Chen Y.. (2016). Genome-wide analysis of the AP2/ERF gene family in physic nut and overexpression of the *JcERF011* gene in rice increased its sensitivity to salinity stress. PLoS ONE 11:e0150879. 10.1371/journal.pone.0150879 26943337 PMC4778941

[B67] Thamilarasan S. K. Park J. I. Jung H. J. Nou I. S. (2014). Genome-wide analysis of the distribution of AP2/ERF transcription factors reveals duplication and CBFs genes elucidate their potential function in *Brassica oleracea*. BMC Genomics 15:422. 10.1186/1471-2164-15-422 24888752 PMC4229850

[B68] Tiwari S. B. Belachew A. Ma S. F. Young M. Ade J. Shen Y.. (2012). The EDLL motif: a potent plant transcriptional activation domain from AP2/ERF transcription factors. Plant J. 70, 855–865. 10.1111/j.1365-313X.2012.04935.x 22321262

[B69] Velivelli S. L. Lojan P. Cranenbrouck S. de Boulois H. D. Suarez J. P. Declerck S.. (2015). The induction of Ethylene response factor 3 (*ERF3*) in potato as a result of co-inoculation with *Pseudomonas* sp. *R41805* and *Rhizophagus irregularis* MUCL 41833-a possible role in plant defense. Plant Signal. Behav. 10:e988076. 10.4161/15592324.2014.988076 25723847 PMC4623016

[B70] Vogel M. O. Moore M. König K. Pecher P. Alsharafa K. Lee J.. (2014). Fast retrograde signaling in response to high light involves metabolite export, *MITOGEN-ACTIVATED PROTEIN KINASE6*, and AP2/ERF transcription factors in Arabidopsis. Plant Cell 26, 1151–1165. 10.1105/tpc.113.121061 24668746 PMC4001375

[B71] Wang L. Wang C. Qin L. Liu W. Wang Y. (2015). *ThERF1* regulates its target genes via binding to a novel cis-acting element in response to salt stress. J. Integr. Plant Biol. 57, 838–847. 10.1111/jipb.12335 25641039

[B72] Wu H. Lv H. Li L. Liu J. Mu S. Li X.. (2015). Genome-Wide Analysis of the AP2/ERF Transcription factors family and the expression patterns of DREB genes in moso bamboo (*Phyllostachys edulis*). PLoS ONE 10:e0126657. 10.1371/journal.pone.0126657 25985202 PMC4436012

[B73] Wuddineh W. A. Mazarei M. Turner G. B. Sykes R. W. Decker S. R. Davis M. F.. (2015). Identification and molecular characterization of the switchgrass AP2/ERF transcription factor superfamily, and overexpression of *PvERF001* for improvement of biomass characteristics for biofuel. Front. Bioeng. Biotechnol. 3:101. 10.3389/fbioe.2015.00101 26258121 PMC4507462

[B74] Xu W. Li F. Ling L. Liu A. (2013). Genome-wide survey and expression profiles of the AP2/ERF family in castor bean (*Ricinus communis* L.). BMC Genomics 14:785. 10.1186/1471-2164-14-785 24225250 PMC4046667

[B75] Zhang C. H. Shangguan L. F. Ma R. J. Sun X. Tao R. Guo L.. (2012). Genome-wide analysis of the AP2/ERF superfamily in peach (*Prunus persica*). Genet. Mol. Res. 11, 4789–4809. 10.4238/2012.October.17.6 23096924

[B76] Zhang L. Li Z. Quan R. Li G. Wang R. Huang R. (2011). An AP2 domain-containing gene, *ESE1*, targeted by the ethylene signaling component EIN3 is important for the salt response in Arabidopsis. Plant Physiol. 157, 854–865. 10.1104/pp.111.179028 21832142 PMC3192559

[B77] Zhang Z. Huang R. (2010). Enhanced tolerance to freezing in tobacco and tomato overexpressing transcription factor *TERF2/LeERF2* is modulated by ethylene biosynthesis. Plant Mol. Biol. 73, 241–249. 10.1007/s11103-010-9609-4 20135196

[B78] Zhao Y. Wei T. Yin K. Q. Chen Z. Gu H. Qu L. J.. (2012). Arabidopsis *RAP2.2* plays an important role in plant resistance to Botrytis cinerea and ethylene responses. New Phytol. 195, 450–460. 10.1111/j.1469-8137.2012.04160.x 22530619

[B79] Zhuang J. Cai B. Peng R. H. Zhu B. Jin X. F. Xue Y.. (2008). Genome-wide analysis of the AP2/ERF gene family in *Populus trichocarpa*. Biochem. Biophys. Res. Commun. 371, 468–474. 10.1016/j.bbrc.2008.04.087 18442469

[B80] Zwack P. J. Compton M. A. Adams C. I. Rashotte A. M. (2016a). Cytokinin response factor 4 (CRF4) is induced by cold and involved in freezing tolerance. Plant Cell Rep. 35, 573–584. 10.1007/s00299-015-1904-8 26650835

[B81] Zwack P. J. De Clercq I. Howton T. C. Hallmark H. T. Hurny A. Keshishian E. A.. (2016b). Cytokinin response factor 6 represses cytokinin-associated genes during oxidative stress. Plant Physiol. 172, 1249–1258. 10.1104/pp.16.00415 27550996 PMC5047073

