# Supplementary material for: Genome-Wide Identification of AP2/ERF Transcription Factors in Cauliflower and Expression Profiling of the ERF Family under Salt and Drought Stresses
Source: Front Plant Sci. 2017 Jun 8;8:946. doi: 10.3389/fpls.2017.00946 (PMC5462956; doi:10.3389/fpls.2017.00946)
Supplement: Supplementary file 1 [file Table1.DOCX]

**Genome-wide identification of AP2/ERF transcription factors in cauliflower and expression profiling of the ERF family under salt and drought stresses**

Hui Li^1, 2^, Yu Wang^1^, Mei Wu^1^, Lihong Li^1^, Cong Li^1^, Zhanpin Han^2^, Jiye Yuan^1^ Chengbin Chen^1^, Wenqin Song^1^, Chunguo Wang^1**^

^1^College of Life Sciences, Nankai University, Tianjin 300071, China;

^2^College of Horticulture and Landscape, Tianjin Agricultural University, Tianjin, 300384, China

**Corresponding author: email: [wangcg@nankai.edu.cn](mailto:wangcg@nankai.edu.cn); Telephone: 86-22-23508241; Fax: 86-22-23508800

Table S1 Primers of 35 AP2/ERF transcription factors of cauliflower used in qRT-PCR

| Primer sequences (5’-3’) |
| --- |
| ABR1a-S: ATAACGCTTTCACGCCTCCCT |
| ABR1a-A: TAGCTGCCTCGACTGCTTTCC |
| AIL6a-S: CGACTCTGCCTCCATAGCCC |
| AIL6a-A: CGCCACAACAGGCTTCTCC |
| AP2/ERF-2S: TCACCCGACTCAATCAACACC |
| AP2/ERF-2A: GCTCCTTGGAACCGAACTCTC |
| CRF2a-S: CAACCAGCACAATCAACAGCC |
| CRF2a-A: CTGCCTCACTCCTCGGTATTTC |
| CRF4a-S: CGTCTACGACAACGCAGCAAT |
| CRF4a-A: TTCTTCCAGGTGCCAGCTTATT |
| CRF6a-S: AACGATACGTGGAGGAGATTAGATT |
| CRF6a-A: AGAGCGTTGTGACCCTTGAGAC |
| ERF001a-S: GCAGCGTTGGCTTACGACAG |
| ERF001a-A: ACCGCCGCCGTTTACAGA |
| ERF003a-S: ACCGCTAAACTCCACAAATGCT |
| ERF003a-A: CCTTCATCGTCTCCGTTACTCC |
| ERF007a-S: CAGCACCGTGAAATCGTGTAGC |
| ERF007a-A: AGTGGAGATCGTCTGCGAATAAGT |
| ERF009a-S: GCAACAACCAACTTCCCTTTCA |
| ERF009a-A: CGATACTCAATCCCATTTCCATT |
| ERF011b-S: AGGACCTACCGCAACGCTCAA |
| ERF011b-A: GCTCCAATAACCCGCCAACAT |
| ERF012b-S: CAGCCAGGGCTTACGATGTTG |
| ERF012b-A: TGATCCGACGGTGACGAGGT |
| ERF016a-S: CTAACGCCGCAGGAGATTCAG |
| ERF016a-A: ATGTTGCTGTAACGAATCCTCCA |
| ERF019-S: AACTTCCCTCACTTGCTTCCTC |
| ERF019-A: TGACCGTCCAAATAATCATACACTG |
| ERF025a-S: TTTGACGTATGTGGCTCCTGCTT |
| ERF025a-A: CGGATTTATCATTCTCGGTGGG |
| ERF034a-S: CCTCGTCCGGTCACTAATTCTC |
| ERF034a-A: TCTCTTCGTCGCTATCTTTGTCC |
| ERF036-S: TTGCCTCTGTCCTCCCTCGTC |
| ERF036-A: CACCCAACAATCCAACGGCTC |
| ERF054a-S: GGACTTTGACGAGGAGCTGAAT |
| ERF054a-A: GTTGAACGTATCCGAAGTGCTG |
| ERF056-S: AACTTCCCAGACATCAAACACG |
| ERF056-A: CCAACTTAGACTCATCCCACGAC |
| ERF069a-S: GCGAAGACGACGAGCAAACAAA |
| ERF069a-A: GCCGGAATCCTGATCTTGAACC |
| ERF071-S: TTTCCCAAACGACGATTCCACC |
| ERF071-A: AACGCCATCAGCTCCTCCGACA |
| ERF088-S: TTTGGCTTATGATGTTGCTGCTC |
| ERF088-A: GGTTCCAAAGAGGCAACAGGG |
| ERF095-S: ACGGTGCTCGTGTCTGGCTT |
| ERF095-A: GCATTCTCGCCATCACCACTT |
| ERF104a-S: GACCAAGCGGCGTTTCAGTT |
| ERF104a-A: ATCCCGTCTCCTGCTCCCAC |
| ERF106a-S: AGAAGAACAGAGGCATTACCGAG |
| ERF106a-A: AGAGTTTCCCTGGCTTCTTTGG |
| ERF109a-S: CGGCGAAGAAAGCAGCG |
| ERF109a-A: GCCCACGGAACCCAATAGC |
| ERF115a-S: CTCGGACCGCTCCCAAT |
| ERF115a-A: TTAGGAGAAATAGGTTGGGTAGAA |
| ERF118a-S: AGACGAGGAGATGGTTGGGTAC |
| ERF118a-A: GTTGAAGAGCCTGAGCACTGAC |
| RAP2-1-S: CGTCTTTGGCTCGGTTCTTACA |
| RAP2-1-A: AGAACAAGCGAGAAGAGCATCAA |
| RAP2-10a-S: ATGGCTTGGCTCTTACTCTACTCC |
| RAP2-10a-A: GCTTCTAACGCATCCACCTGA |
| RAP2-11b-S: CGTTGGAGTCAGGCAAAGGC |
| RAP2-11b-A: GGTTAGCGAAATTGGTGCGAGTA |
| RAP2-12-S: AGGAGAACCCACCTGCCAAG |
| RAP2-12-A: AACAACCGAATGAGTTACTACCCTG |
| RAP2-4a-S: CGGCTTAACTTCCCTAACCTGC |
| RAP2-4a-A: TGCTTTCGTCGTCTTCTCCTGT |
| RAP2-7a-S: GAGGAGGGATGGTGAGTAACTGG |
| RAP2-7a-A: AGAGGCATTGTCATTGGGAGGT |
| RAV2a-S: GAGTTTGGCTTGGCACTTTCAG |
| RAV2a-A: GTATTCGCTCCGTTGCGTTTAT |
