# Supplementary material for: Genome-Wide Identification of AP2/ERF Transcription Factors in Cauliflower and Expression Profiling of the ERF Family under Salt and Drought Stresses
Source: Front Plant Sci. 2017 Jun 8;8:946. doi: 10.3389/fpls.2017.00946 (PMC5462956; doi:10.3389/fpls.2017.00946)
Supplement: Supplementary file 3 [file Table3.docx]

**Genome-wide identification** **of AP2/ERF transcription factors in cauliflower and expression profiling of the ERF family under salt and drought stresses**

Hui Li^1, 2^, Yu Wang^1^, Mei Wu^1^, Lihong Li^1^, Cong Li^1^, Zhanpin Han^2^, Jiye Yuan^1^ Chengbin Chen^1^, Wenqin Song^1^, Chunguo Wang^1**^

^1^College of Life Sciences, Nankai University, Tianjin 300071, China;

^2^College of Horticulture and Landscape, Tianjin Agricultural University, Tianjin, 300384, China

**Corresponding author: email: [wangcg@nankai.edu.cn](mailto:wangcg@nankai.edu.cn); Telephone: 86-22-23508241; Fax: 86-22-23508800

**Table S3** The relative expression levels of 35 cauliflower AP2/ERF transcription factors under salt stress.

| Genes | Relative expression level | | | | | | | |
| --- | --- | --- | --- | --- | --- | --- | --- | --- |
|  | 0h | SD (0h) | 4h | SD (4h) | 8h | SD (8h) | 24h | SD (24h) |
| Bra-botrytis-ABR1a | 0.0901 | 0.00193 | 0.032 | 0.00078 | 0.064 | 0.00103 | 1.28 | 0.02337 |
| Bra-botrytis-AIL6a | 0.1132 | 0.00371 | 0.461 | 0.01436 | 0.186 | 0.00475 | 1.368 | 0.02306 |
| Bra-botrytis-AP2/ERF-2 | 0.1421 | 0.03607 | 0.638 | 0.02073 | 1 | 0.02567 | 0.151 | 0.0017 |
| Bra-botrytis-CRF2a | 0.0648 | 0.00154 | 0.118 | 0.00281 | 0.216 | 0.00378 | 1.217 | 0.04406 |
| Bra-botrytis-CRF4a | 0.1132 | 0.00124 | 1.872 | 0.04091 | 0.314 | 0.01022 | 0.819 | 0.02821 |
| Bra-botrytis-CRF6a | 0.401 | 0.01019 | 0.724 | 0.01735 | 0.271 | 0.00271 | 1.468 | 0.01277 |
| Bra-botrytis-ERF001a | 0.0513 | 0.00047 | 0.436 | 0.01003 | 0.09 | 0.00116 | 0.51 | 0.00429 |
| Bra-botrytis-ERF003a | 0.0102 | 0.00018 | 0.03 | 0.00035 | 0.289 | 0.00619 | 1.303 | 0.03163 |
| Bra-botrytis-ERF007a | 0.1041 | 0.01052 | 0.879 | 0.0188 | 0.606 | 0.01085 | 1.516 | 0.00394 |
| Bra-botrytis-ERF009a | 0.083 | 0.00082 | 1.254 | 0.00323 | 0.195 | 0.00297 | 1.347 | 0.02025 |
| Bra-botrytis-ERF011b | 0.034 | 0.00097 | 0.393 | 0.00455 | 0.143 | 0.00252 | 1.266 | 0.0156 |
| Bra-botrytis-ERF012b | 0.0107 | 0.00096 | 0.015 | 0.00032 | 0.059 | 0.00112 | 1.413 | 0.0985 |
| Bra-botrytis-ERF016a | 0.0384 | 0.00085 | 0.139 | 0.00176 | 0.012 | 0.0002 | 1.331 | 0.01201 |
| Bra-botrytis-ERF019 | 0.0042 | 0.00009 | 0.029 | 0.00028 | 0.026 | 0.00056 | 1.203 | 0.00432 |
| Bra-botrytis-ERF025a | 0.009 | 0.00018 | 0.256 | 0.00736 | 0.008 | 0.00033 | 1.363 | 0.02031 |
| Bra-botrytis-ERF034a | 0.397 | 0.00495 | 0.404 | 0.01128 | 0.417 | 0.0097 | 1.562 | 0.01985 |
| Bra-botrytis-ERF036 | 0.1151 | 0.01971 | 0.078 | 0.00247 | 0.033 | 0.00081 | 1.817 | 0.02728 |
| Bra-botrytis-ERF054a | 0.1364 | 0.01692 | 0.192 | 0.00684 | 1 | 0.0286 | 1.333 | 0.02531 |
| Bra-botrytis-ERF056 | 0.2157 | 0.01762 | 0.912 | 0.03447 | 1 | 0.02281 | 1.023 | 0.02057 |
| Bra-botrytis-ERF069a | 0.699 | 0.00948 | 0.767 | 0.02974 | 0.586 | 0.0124 | 1.436 | 0.04061 |
| Bra-botrytis-ERF071 | 0.3162 | 0.0175 | 0.741 | 0.02501 | 0.562 | 0.01375 | 1.579 | 0.03732 |
| Bra-botrytis-ERF088 | 0.069 | 0.00627 | 0.573 | 0.00696 | 0.047 | 0.00086 | 1.431 | 0.04963 |
| Bra-botrytis-ERF095 | 0.076 | 0.00681 | 0.465 | 0.00539 | 0.163 | 0.0031 | 1.642 | 0.05265 |
| Bra-botrytis-ERF104a | 0.0258 | 0.00517 | 0.149 | 0.0021 | 0.033 | 0.00072 | 1.411 | 0.04839 |
| Bra-botrytis-106a | 0.077 | 0.00666 | 1.178 | 0.06114 | 1 | 0.01552 | 0.212 | 0.00868 |
| Bra-botrytis-ERF109a | 0.069 | 0.01144 | 0.095 | 0.00095 | 0.005 | 0.00011 | 1.384 | 0.05447 |
| Bra-botrytis-ERF115a | 0.089 | 0.01057 | 0.234 | 0.00654 | 1.144 | 0.0418 | 0.237 | 0.0074 |
| Bra-botrytis-ERF118a | 0.804 | 0.00699 | 2.153 | 0.02072 | 0.793 | 0.02994 | 0.471 | 0.00634 |
| Bra-botrytis-RAP2-1 | 0.197 | 0.00609 | 0.729 | 0.00666 | 1.144 | 0.04348 | 0.618 | 0.01411 |
| Bra-botrytis-RAP2-10a | 0.679 | 0.00607 | 0.541 | 0.00794 | 0.801 | 0.03512 | 1 | 0.01347 |
| Bra-botrytis-RAP2-11b | 0.069 | 0.00975 | 0.149 | 0.00139 | 0.071 | 0.00217 | 1 | 0.02368 |
| Bra-botrytis-RAP2-12 | 0.846 | 0.01343 | 0.514 | 0.00205 | 0.563 | 0.00963 | 1.107 | 0.01183 |
| Bra-botrytis-RAP2-4a | 0.8 | 0.00793 | 0.544 | 0.00303 | 0.518 | 0.00892 | 1.216 | 0.0105 |
| Bra-botrytis-RAP2-7a | 0.102 | 0.01062 | 0.795 | 0.00563 | 0.534 | 0.00785 | 1.268 | 0.01508 |
| Bra-botrytis-RAV2a | 0.846 | 0.0539 | 1.136 | 0.00893 | 0.45 | 0.0102 | 1.605 | 0.01172 |

Notes: SD =standard deviation.
