# Supplementary material for: Genome-Wide Identification of AP2/ERF Transcription Factors in Cauliflower and Expression Profiling of the ERF Family under Salt and Drought Stresses
Source: Front Plant Sci. 2017 Jun 8;8:946. doi: 10.3389/fpls.2017.00946 (PMC5462956; doi:10.3389/fpls.2017.00946)
Supplement: Supplementary file 4 [file Table4.docx]

**Genome-wide identification** **of AP2/ERF transcription factors in cauliflower and expression profiling of the ERF family under salt and drought stresses**

Hui Li^1, 2^, Yu Wang^1^, Mei Wu^1^, Lihong Li^1^, Cong Li^1^, Zhanpin Han^2^, Jiye Yuan^1^ Chengbin Chen^1^, Wenqin Song^1^, Chunguo Wang^1**^

^1^College of Life Sciences, Nankai University, Tianjin 300071, China;

^2^College of Horticulture and Landscape, Tianjin Agricultural University, Tianjin, 300384, China

**Corresponding author: email: [wangcg@nankai.edu.cn](mailto:wangcg@nankai.edu.cn); Telephone: 86-22-23508241; Fax: 86-22-23508800

**Table S4** The relative expression levels of 35 cauliflower AP2/ERF transcription factors under drought stress.

| Genes | Relative expression level | | | | | | | |
| --- | --- | --- | --- | --- | --- | --- | --- | --- |
|  | 0h | SD (0h) | 4h | SD (4h) | 8h | SD (8h) | 24h | SD (24h) |
| Bra-botrytis-ABR1a | 0.0901 | 0.00193 | 0.006 | 0.00012 | 0.039 | 0.00068 | 2.9 | 0.14302 |
| Bra-botrytis-AIL6a | 0.1132 | 0.00371 | 0.191 | 0.00733 | 0.274 | 0.00557 | 0.296 | 0.01809 |
| Bra-botrytis-AP2/ERF-2 | 0.1421 | 0.03607 | 0.61 | 0.01531 | 0.507 | 0.01212 | 0.739 | 0.03552 |
| Bra-botrytis-CRF2a | 0.0648 | 0.00154 | 0.22 | 0.00514 | 0.656 | 0.01709 | 2.9 | 0.14932 |
| Bra-botrytis-CRF4a | 0.1132 | 0.00124 | 0.012 | 0.00041 | 0.014 | 0.00029 | 0.111 | 0.00563 |
| Bra-botrytis-CRF6a | 0.401 | 0.01019 | 0.742 | 0.02456 | 1 | 0.01668 | 2.35 | 0.1414 |
| Bra-botrytis-ERF001a | 0.0513 | 0.00047 | 0.007 | 0.00007 | 0.022 | 0.0004 | 0.55 | 0.02598 |
| Bra-botrytis-ERF003a | 0.0102 | 0.00018 | 0.067 | 0.00241 | 0.08 | 0.00094 | 0.039 | 0.00152 |
| Bra-botrytis-ERF007a | 0.1041 | 0.01052 | 0.427 | 0.01446 | 0.739 | 0.00535 | 0.742 | 0.02941 |
| Bra-botrytis-ERF009a | 0.083 | 0.00082 | 0.065 | 0.00301 | 0.372 | 0.00457 | 0.61 | 0.02463 |
| Bra-botrytis-ERF011b | 0.034 | 0.00097 | 0.677 | 0.02173 | 0.95 | 0.01016 | 1.618 | 0.06387 |
| Bra-botrytis-ERF012b | 0.0107 | 0.00096 | 0.015 | 0.00073 | 0.123 | 0.00358 | 0.639 | 0.0328 |
| Bra-botrytis-ERF016a | 0.0384 | 0.00085 | 0.028 | 0.00113 | 0.286 | 0.00872 | 1.391 | 0.09842 |
| Bra-botrytis-ERF019 | 0.0042 | 0.00009 | 0.002 | 0.00008 | 0.026 | 0.00035 | 1.579 | 0.07458 |
| Bra-botrytis-ERF025a | 0.009 | 0.00018 | 0.008 | 0.00012 | 0.066 | 0.00089 | 1.685 | 0.01546 |
| Bra-botrytis-ERF034a | 0.397 | 0.00495 | 1 | 0.00681 | 0.778 | 0.0104 | 0.553 | 0.01514 |
| Bra-botrytis-ERF036 | 0.1151 | 0.01971 | 0.045 | 0.00033 | 0.192 | 0.00348 | 0.228 | 0.00406 |
| Bra-botrytis-ERF054a | 0.1364 | 0.01692 | 0.695 | 0.00494 | 0.973 | 0.01647 | 1.314 | 0.02864 |
| Bra-botrytis-ERF056 | 0.2157 | 0.01762 | 0.859 | 0.01427 | 0.747 | 0.01373 | 1.063 | 0.00976 |
| Bra-botrytis-ERF069a | 0.699 | 0.00948 | 0.864 | 0.00782 | 1.046 | 0.01512 | 1.491 | 0.01804 |
| Bra-botrytis-ERF071 | 0.3162 | 0.0175 | 0.72 | 0.0084 | 0.843 | 0.00972 | 1.469 | 0.01422 |
| Bra-botrytis-ERF088 | 0.069 | 0.00627 | 0.081 | 0.00136 | 0.279 | 0.00838 | 2.447 | 0.05594 |
| Bra-botrytis-ERF095 | 0.076 | 0.00681 | 0.094 | 0.0039 | 0.113 | 0.00638 | 3.511 | 0.10818 |
| Bra-botrytis-ERF104a | 0.0258 | 0.00517 | 0.075 | 0.00102 | 0.531 | 0.00602 | 4.346 | 0.11865 |
| Bra-botrytis-106a | 0.077 | 0.00666 | 0.05 | 0.00104 | 0.158 | 0.00316 | 0.434 | 0.01151 |
| Bra-botrytis-ERF109a | 0.069 | 0.01144 | 0.005 | 0.00004 | 0.071 | 0.00087 | 4.346 | 0.09955 |
| Bra-botrytis-ERF115a | 0.089 | 0.01057 | 0.108 | 0.00281 | 0.117 | 0.00432 | 0.67 | 0.02387 |
| Bra-botrytis-ERF118a | 0.804 | 0.00699 | 0.898 | 0.0199 | 1.742 | 0.03468 | 4.346 | 0.09816 |
| Bra-botrytis-RAP2-1 | 0.197 | 0.00609 | 0.464 | 0.00252 | 0.663 | 0.01226 | 2.099 | 0.02321 |
| Bra-botrytis-RAP2-10a | 0.679 | 0.00607 | 0.81 | 0.01296 | 1 | 0.01342 | 1.804 | 0.03159 |
| Bra-botrytis-RAP2-11b | 0.069 | 0.00975 | 0.088 | 0.00112 | 0.198 | 0.00298 | 2.099 | 0.04065 |
| Bra-botrytis-RAP2-12 | 0.846 | 0.01343 | 0.512 | 0.00567 | 0.907 | 0.01275 | 2.099 | 0.02572 |
| Bra-botrytis-RAP2-4a | 0.8 | 0.00793 | 0.75 | 0.01361 | 1 | 0.01045 | 1.955 | 0.03438 |
| Bra-botrytis-RAP2-7a | 0.102 | 0.01062 | 0.234 | 0.00125 | 0.367 | 0.00346 | 0.424 | 0.00526 |
| Bra-botrytis-RAV2a | 0.846 | 0.0539 | 1.069 | 0.01789 | 0.9524 | 0.00689 | 2.158 | 0.17251 |

Notes: SD =standard deviation.
