# Supplementary material for: Genome-Wide Identification of AP2/ERF Transcription Factors in Cauliflower and Expression Profiling of the ERF Family under Salt and Drought Stresses
Source: Front Plant Sci. 2017 Jun 8;8:946. doi: 10.3389/fpls.2017.00946 (PMC5462956; doi:10.3389/fpls.2017.00946)
Supplement: Supplementary file 5 [file Presentation1.pdf]

# Genome-wide identification of AP2/ERF transcription factors in cauliflower and expression profiling of the ERF family under salt and drought stresses

Hui Li[1, 2], Yu Wang[1], Mei Wu[1], Lihong Li[1], Cong Li[1], Zhanpin Han[2], Jiye Yuan[1] Chengbin Chen[1], Wenqin Song[1], Chunguo Wang[1**]

[1]College of Life Sciences, Nankai University, Tianjin 300071, China;

[2]College of Horticulture and Landscape, Tianjin Agricultural University, Tianjin, 300384, China

**Corresponding author: email: wangcg@nankai.edu.cn; Telephone: 86-22-23508241; Fax: 86-22-23508800

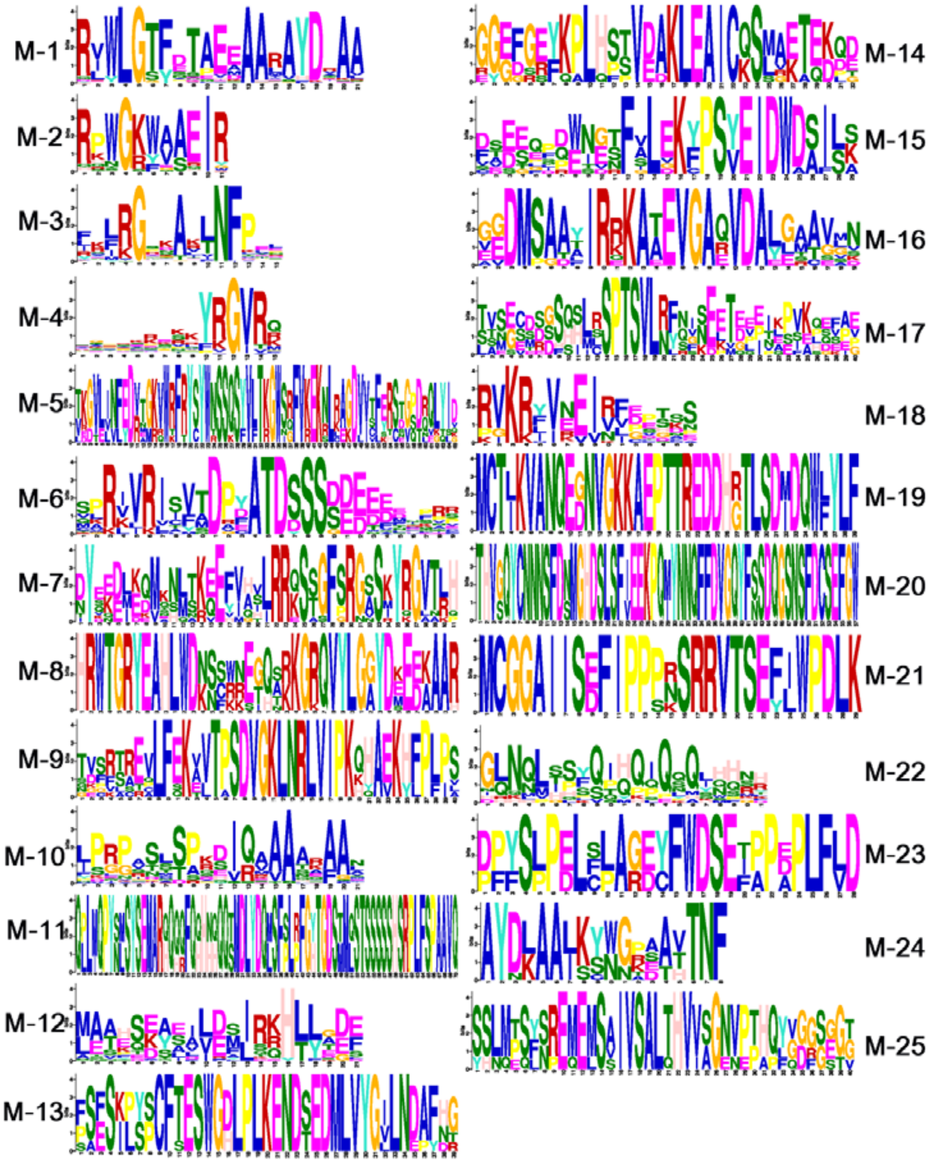


**Figure S1** Sequence logos of 25 conserved motifs within AP2/ERF transcription factors of cauliflower. The overall height of the stack indicated the sequence conservation. The height of residues within the stack indicates the relative frequency of each residue at that position.

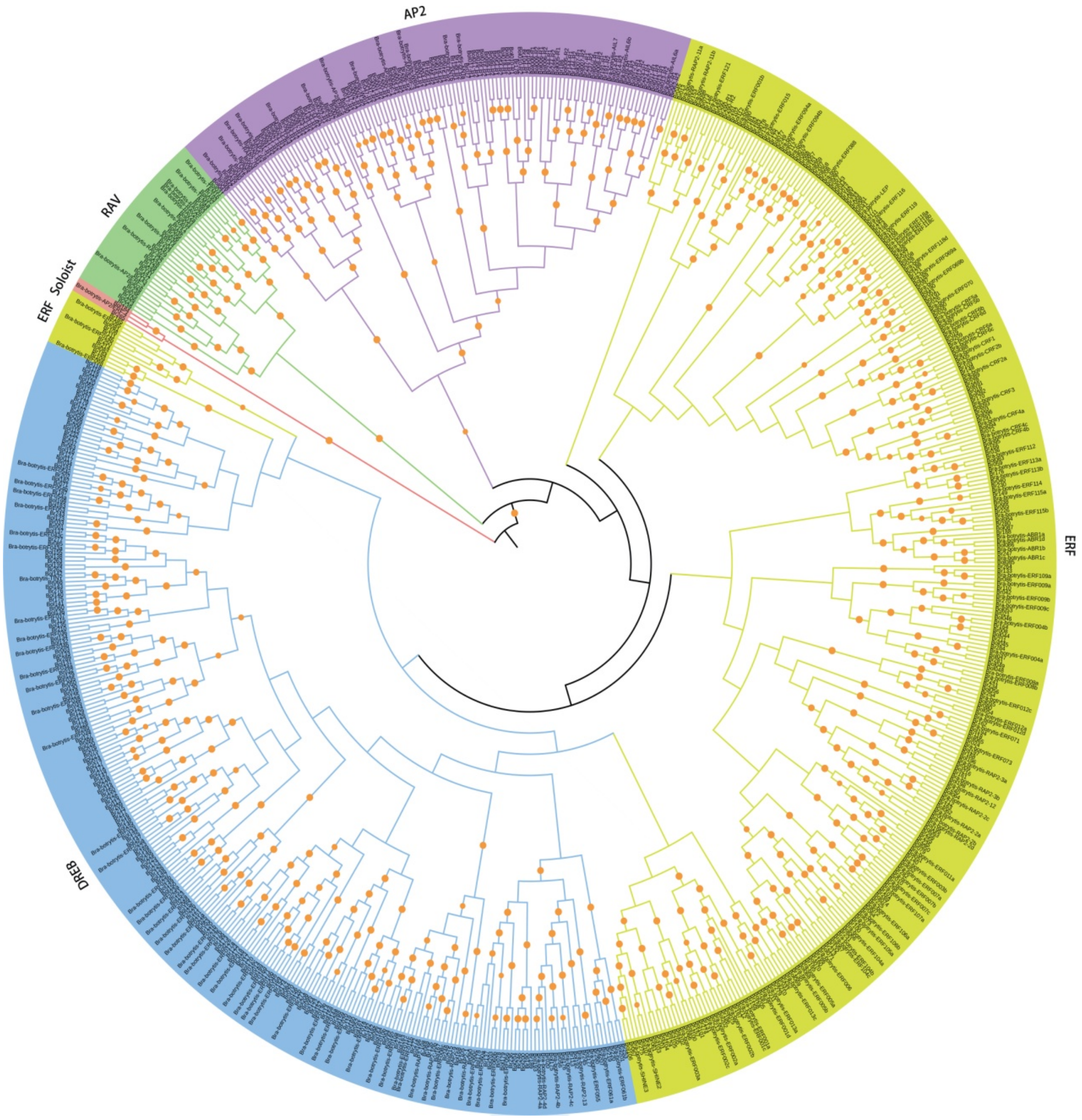


**Figure S2** Phylogenetic tree of AP2/ERF TFs in cauliflower, cabbage and Chinese cabbage. AP2, RAV and the Soloist families, ERF and DREB subfamilies were marked with different colors, respectively. Bootstrap values over 80% were showed by dots with different size. The protein sequences of AP2/ERF TFs in cabbage were referenced to the data from Thamilarasan et al., 2014 (BMC Genomics 15, 422); The protein sequences of AP2/ERF TFs Chinese cabbage referenced to the data from Song et al., 2013 (BMC Genomics 14, 573).
